# How Can Phytoplankton Pigments Be Best Used to Characterize Surface Ocean Phytoplankton Groups for Ocean Color Remote Sensing Algorithms?

**DOI:** 10.1029/2019JC015604

**Published:** 2019-11-11

**Authors:** Sasha J. Kramer, David A. Siegel

**Affiliations:** ^1^ Interdepartmental Graduate Program in Marine Science University of California Santa Barbara CA USA; ^2^ Earth Research Institute University of California Santa Barbara CA USA; ^3^ Department of Geography University of California Santa Barbara CA USA

**Keywords:** phytoplankton, HPLC pigments, remote sensing

## Abstract

High‐performance liquid chromatography (HPLC) remains one of the most widely applied methods for estimation of phytoplankton community structure from ocean samples, which are used to create and validate satellite retrievals of phytoplankton community structure. HPLC measures the concentrations of phytoplankton pigments, some of which are useful chemotaxonomic markers for phytoplankton groups. Here, consistent suites of HPLC phytoplankton pigments measured on global surface water samples are compiled across spatial scales. The global dataset includes >4,000 samples from every major ocean basin and representing a wide range of ecological regimes. The local dataset is composed of six time series from long‐term observatory sites. These samples are used to quantify the potential and limitations of HPLC for understanding surface ocean phytoplankton groups. Hierarchical cluster and empirical orthogonal function analyses are used to examine the associations between and among groups of phytoplankton pigments and to diagnose the main controls on these associations. These methods identify four major groups of phytoplankton on global scales (cyanobacteria, diatoms/dinoflagellates, haptophytes, and green algae) that can be identified by diagnostic biomarker pigments. On local scales, the same methods identify more and different taxonomic groups of phytoplankton than are detectable in the global dataset. Notably, diatom and dinoflagellate pigments group together on global scales, but dinoflagellate marker pigments always separate from diatoms on local scales. Together, these results confirm that HPLC pigments can be used for satellite algorithm quantification of no more than four phytoplankton groups on global scales, but can provide higher resolution for local‐scale algorithm development and validation.

## Key Points

- Statistical methods are applied to phytoplankton pigment data to assess their utility in defining phytoplankton groups for remote sensing algorithms
- Four taxonomically distinct phytoplankton groups are identified on global scales while often more and different groups are found on local scales
- The potential and limitations of HPLC pigments for remote sensing algorithm validation to characterize phytoplankton across spatial scales are considered

## 1 Introduction

Phytoplankton form the base of the marine food web and are essential to biogeochemical cycling as a source of elemental compounds and nutrients (e.g., Le Quéré et al., 2005). In order to quantify the ecological, biogeochemical, and economic importance of phytoplankton in the global ocean, it is necessary to accurately describe the distribution and abundance of various taxonomic groups (Falkowski & Oliver, 2007; Guidi et al., 2009; Legendre, 1990). The global surface ocean distribution of total chlorophyll‐*a*, which is often used as a proxy for phytoplankton biomass, has been well described using satellite‐based methods (e.g., Martinez et al., 2009; Siegel et al., 2013). However, progress toward a unified satellite‐based approach for assessing the phytoplankton groups that comprise the total chlorophyll‐*a* distribution is ongoing (IOCCG, 2014 and references therein). National Aeronautics and Space Administration (NASA)'s upcoming hyperspectral Plankton, Aerosol, Cloud and ocean Ecosystem (PACE) mission will provide unprecedented spectral resolution and thus offers the potential for new insights into phytoplankton community dynamics on local to global scales (Werdell et al., 2019). In anticipation of PACE, there will likely be an increase in algorithms to detect phytoplankton groups from ocean color remote sensing (Catlett & Siegel, 2018; Chase et al., 2017).

The taxonomic diversity of phytoplankton is often simplified into functional groups based on their ecological roles and physiological traits (Le Quéré et al., 2005). Phytoplankton Functional Types (PFTs) seek to quantify specific phytoplankton groups based on their roles in elemental cycling and the group's cell size. This designation of PFTs broadly corresponds to specific taxonomic groups: for instance, diatoms are microsized and nanosized phytoplankton that require siliceous nutrients and are thought to dominate export production. Conversely, haptophytes are nanosized to picosized phytoplankton that include both dimethyl sulfide‐ (e.g., *Phaeocystis* spp.) and calcium carbonate‐producers (e.g., *Emiliania huxleyi*). Finally, cyanobacteria are picosized bacterioplankton that make important contributions to global primary production (e.g., *Synechococcus* and *Prochlorococcus* spp.).

There are many existing methods to measure and describe phytoplankton taxonomy and functional diversity, including microscopy, optical proxies, quantitative cell imaging, and genomic sequencing, each with associated strengths and weaknesses. While microscopy and quantitative imaging remain the “gold standard” for phytoplankton identification, high‐performance liquid chromatography (HPLC) remains one of the most widespread, methodical, and quality‐controlled methods currently available (Van Heukelem & Hooker, 2011). HPLC enables the determination of the concentrations of ~25 phytoplankton pigments, some of which are useful chemotaxonomic markers for specific phytoplankton groups either in their presence or in their co‐occurrence with other phytoplankton pigments. The difficulty is that many if not most phytoplankton pigments are shared among taxonomic groups (Table 1, following Jeffrey et al., 2011 and references therein), making chemotaxonomic quantification of phytoplankton groups challenging. Fortunately, groups with similar evolutionary lineages naturally tend to share the same groups of pigments (e.g., Falkowski et al., 2004). Red algae (diatoms, dinoflagellates, haptophytes, and cryptophytes) have more pigments in common with each other than with green algae or cyanobacteria. These taxonomic groups can be broadly separated into size classes, using methods that relate biomarker pigments to size relying on general relationships between phytoplankton groups and cell size.

**Table 1:** Summary of 18 Pigments Used in This Analysis (17 Accessory Pigments and Monovinyl Chlorophyll‐*a*) and the Distribution of These Pigments Across 12 Taxonomic Groups, Including the Four Major Taxonomic Groups Identified in This Analysis (Diatoms and Dinoflagellates, Haptophytes, Green Algae, and Cyanobacteria)

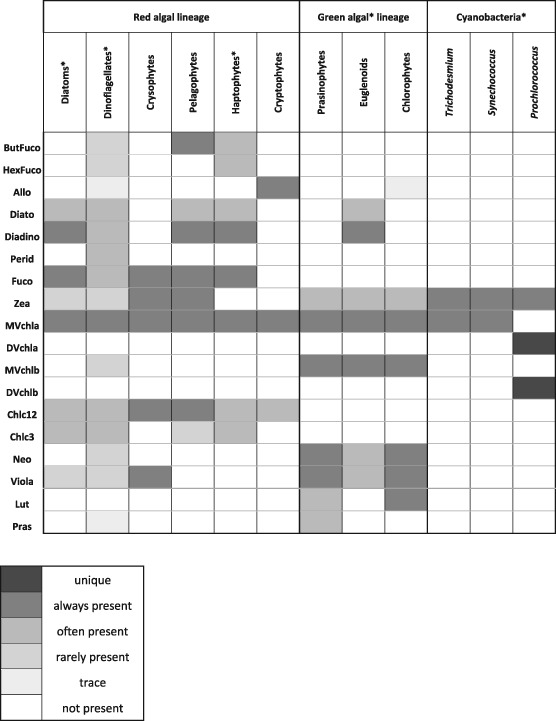

*Note*. Known distributions of each pigment in each group (for the species in each group that have been cultured and had HPLC analysis performed) are shown (adapted from Jeffrey et al., 2011 and references therein). Stars indicate the major taxa identified in this analysis.

Abbreviation: HPLC: high‐performance liquid chromatography.

Pigment‐based methods for characterizing phytoplankton community structure are limited by the variable occurrence and plasticity of pigments across species, groups, strains, and environmental conditions (Table 1). Changes in pigment composition and concentration (and thus ratios of pigments to total chlorophyll‐*a* concentration or phytoplankton carbon biomass) may not occur linearly with changes in the environment. Intercellular pigment concentrations will be highly susceptible to light, nutrient, and temperature variations; species‐specific and strain‐specific compositional variations are also found (Havskum et al., 2004; Schlüter et al., 2000). Likewise, measured changes in pigment composition and concentration in one species are not easily transferred between and among other strains of the same species (Irigoien et al., 2004; Zapata et al., 2004).

Despite these challenges, HPLC pigment data remain in wide use for characterizing phytoplankton groups on local to global scales, particularly for the calibration and validation of ocean color remote sensing algorithms. Many analytical approaches have been developed for this purpose. Some of these methods use weighted contributions of pigments to total chlorophyll‐*a* while other methods rely on threshold ratio values of specific pigments to diagnose the dominance of a given group. For instance, in the Diagnostic Pigment Analysis (DPA; Claustre, 1994; Uitz et al., 2006), certain pigments are used to represent groups of phytoplankton that contribute to each of three phytoplankton size classes. Hierarchical cluster and empirical orthogonal function (EOF) analyses (C. R. Anderson, Siegel, et al., 2008; Catlett & Siegel, 2018; Latasa & Bidigare, 1998) seek to group pigments based on the correlation and co‐occurrence between and among HPLC pigments. The matrix inversion method CHEMTAX (Mackey et al., 1996) assumes that pigment ratios are known for each phytoplankton group and that linear relationships exist among phytoplankton pigment ratios for a given data set. Under these assumptions, the contribution of each group to total chlorophyll‐*a* can be determined. However, CHEMTAX results are often sensitive to choice of pigment ratios and will not be used here (e.g., Latasa, 2007; Pan et al., 2011; Swan et al., 2016).

The development of robust global algorithms to derive phytoplankton community composition from satellite ocean color has long been a research community‐wide goal [i.e., IOCCG, 2014; Bracher et al., 2017]. Such algorithms would allow for global estimates of phytoplankton groups on broader spatiotemporal scales than currently exist and would support many applications, from assessment of export fluxes to fisheries management (e.g., Fogarty et al., 2016; Bisson et al., 2018; etc.). The development and validation of these algorithms requires determinations of surface ocean phytoplankton community composition on both global and local scales; HPLC pigments remain the only data source widely sampled, standardized, and available for this purpose. Hence, understanding the variability of these data on global scales is a first step for developing robust satellite algorithms to quantify phytoplankton groups.

Here, a compilation of consistent surface ocean HPLC pigment observations is constructed and used to quantify the potential and limitations of using HPLC pigments to assess global and local surface ocean phytoplankton community structure. Results are shown for statistical analyses using HPLC pigment observations (hierarchical clustering and EOFs) on both local and global scales, to identify groups of pigments that are representative of specific groups of phytoplankton. By examining the composition and average concentration of pigments within each group and across the statistical methods used, the distributions of phytoplankton groups can be interpreted. The present results describe robust patterns in four major taxonomic groups on global scales (cyanobacteria, diatoms and dinoflagellates, haptophytes, and green algae). On local scales, HPLC pigments can characterize up to six phytoplankton groups, which are more often than not different from those identified globally. While the taxonomic utility of pigment‐based approaches can be limited, the results shown here suggest that HPLC pigments are well suited to calibration and validation of global remote sensing applications that will identify these same four groups on global scales, while the development of regional remote sensing algorithms remains important to maximize local scale information and distinguish higher resolution taxonomic features.

## 2 Materials and Methods

### 2.1 HPLC Pigment Data

**Figure 1:**
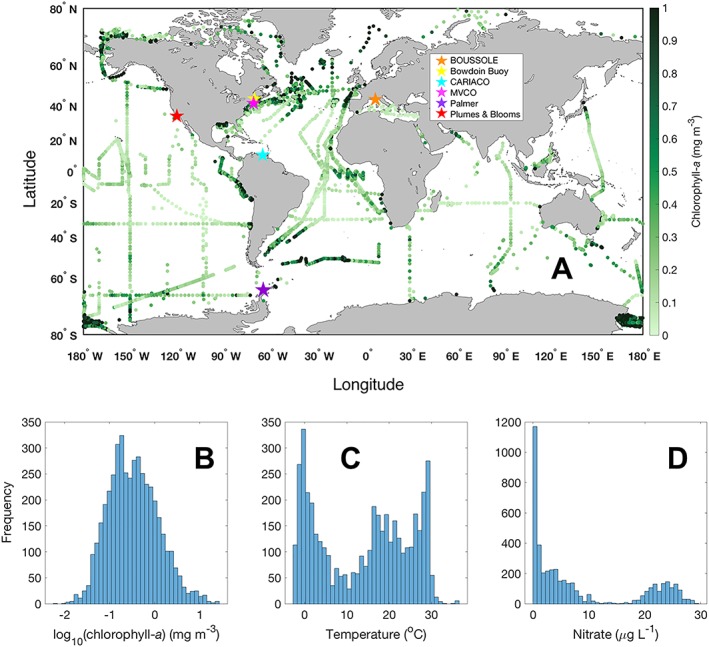
(a) Total chlorophyll‐*a* concentration for all samples in the global analysis (in green, *N* = 4,480). Values greater than 1 mg/m^3^ are colored as equal to 1 mg/m^3^. Local observatory sites are also shown: BOUSSOLE (orange star), Bowdoin Buoy (yellow star), CARIACO (cyan star), MVCO (pink star), Palmer LTER (purple star), and Plumes and Blooms (red star). Histograms show the frequency distribution of (B) log_10_(chlorophyll‐*a*), (c) temperature, and (d) annual mean nitrate concentration for the global dataset used in this analysis.

The present analysis requires synthesis of HPLC phytoplankton pigment surface samples with geographic diversity, with the same pigments measured for all cruises, and from labs with quality assurance protocols in place. The global dataset was constructed from near‐surface HPLC phytoplankton pigment observations, which were compiled from 66 oceanographic research cruises conducted between 2000 and 2018 (Table S1). The dataset includes samples from the Atlantic, Pacific, Indian, Arctic, and Southern Oceans for both coastal and open ocean sites, over a broad range of chlorophyll‐*a* concentrations from oligotrophic to eutrophic conditions (Figures 1a and 1b). For each sample in the global dataset, the full pigment suite is supplemented with measurements of latitude, longitude, date and time, sampling depth, water temperature, salinity, annually averaged mean nitrate concentration, and water depth (data sources in Table S1). In the event of replicate samples in space or time, an average of the replicates was used.

Strict criteria are applied to reduce potential sources of uncertainty: (1) As this dataset aims to support remote sensing applications, all samples used in this analysis were taken in the surface ocean from a depth of 7 meters or shallower; (2) HPLC data were analyzed at one of six labs (see *Quality assurance and quality control*, below); (3) A consistent suite of 25 pigments was measured. These pigments (and their abbreviation herein) include total chlorophyll‐*a* (Tchla, the sum of monovinyl chlorophyll‐*a*, divinyl chlorophyll a, chlorophyllide, and chlorophyll‐*a* allomers and epimers), total chlorophyll b (Tchlb, the sum of monovinyl chlorophyll b, divinyl chlorophyll b, and chlorophyll b epimers), total chlorophyll c (Tchlc, the sum of chlorophylls c1, c2, and c3), alpha‐beta carotene (ABcaro, the sum of alpha and beta carotenes), 19′‐hexanoyloxyfucoxanthin (HexFuco), 19′‐butanoyloxyfucoxanthin (ButFuco), alloxanthin (Allo), fucoxanthin (Fuco), peridinin (Perid), diatoxanthin (Diato), diadinoxanthin (Diadino), zeaxanthin (Zea), monovinyl chlorophyll‐*a* (MVchla), divinyl chlorophyll a (DVchla), chlorophyllide (chllide), monovinyl chlorophyll b (MVchlb), divinyl chlorophyll b (DVchlb), chlorophyll c_1_+c_2_ (Chlc12), chlorophyll c_3_ (Chlc3), lutein (Lut), neoxanthin (Neo), violaxanthin (Viola), phaeophytin (Phytin), phaeophorbide (Phide), prasinoxanthin (Pras). Datasets that did not measure either or both of the divinyl chlorophylls (which are essential for separating *Prochlorococcus* from *Synechococcus*) or did not separate lutein (which is found in only green algae) and zeaxanthin (which is found in red algae, green algae, and cyanobacteria) were not included in the final dataset. Taken together, these criteria eliminated many datasets from consideration that were included in previous global summaries [cf., Uitz et al., 2006; Peloquin et al., 2013; Swan et al., 2016]. Further, all degradation pigments (chllide, Phytin, and Phide) were removed from all further analysis as well as redundant calculated values (MVchla, Tchlb, Tchlc, and ABcaro), leaving seventeen accessory pigments and Tchla. The chemotaxonomic utility of the remaining pigments used in statistical analyses is illustrated in Table 1. This table, adapted from Jeffrey et al. (2011) and references therein, describes many (but certainly not all) possible pigment compositions for a given taxonomic group of phytoplankton.

To supplement the global dataset, a suite of local datasets were constructed from time series observatory sites where HPLC phytoplankton pigments were consistently measured (locations are stars in Figure 1a). The selected time series sites are: Martha′s Vineyard Coastal Observatory (MVCO); BOUée pour l′acquiSition d′une Série Optique à Long termE (BOUSSOLE), French Mediterranean Sea; CArbon Retention In A Colored Ocean (CARIACO), Cariaco Basin; Palmer Long Term Ecological Research Program (LTER), West Antarctic Peninsula; Bowdoin College Buoy, Gulf of Maine; and Plumes and Blooms, Santa Barbara Channel. The same criteria were applied to the local data for consistency with the global data: only surface samples were considered in this analysis, a complete pigment suite was measured for each sample, and the samples were analyzed at the facilities listed below. The local dataset was not included in the global summaries of total chlorophyll‐*a* concentration, temperature, and nitrate concentration (Figures 1b–1d) given the large dynamic range in these parameters over a seasonal cycle of sampling.

### 2.2 Quality Assurance and Quality Control

Precautions were taken to remove potential sources of uncertainty from this global dataset by assuring the quality of the samples used here, as HPLC is a highly sensitive and variable analysis (Van Heukelem & Hooker, 2011). First, only HPLC data that had been processed at any one of six labs was included in the global dataset: Horn Point Laboratory (HPL), NASA Goddard Space Flight Center (NASA GSFC), Laboratoire Oceanographique de Villefranche‐sur‐Mer (LOV), the Australian Commonwealth Scientific and Industrial Research Organisation (CSIRO), the Alfred Wegner Institute (AWI), and the DiTullio lab at the College of Charleston (Figure S1 in the supporting information). Four of these six laboratories (HPL, NASA GSFC, LOV, and CSIRO) participated in the NASA SeaWiFS HPLC Analysis Round‐Robin Experiments (SeaHARRE, Hooker et al., 2012). The other two labs use common approaches for HPLC methodology, both of which were evaluated through the SeaHARRE process: the Barlow et al. (1997) method (AWI) and the Zapata et al. (2000) method (DiTullio). The influence of data source was examined using a dummy control in the statistical analyses to follow. Time series samples were also processed at one of the above six labs with the exception of the Palmer LTER; Palmer HPLC pigments were measured at Rutgers University using the Wright et al. (1991) method, which was also evaluated through SeaHARRE.

**Figure 2:**
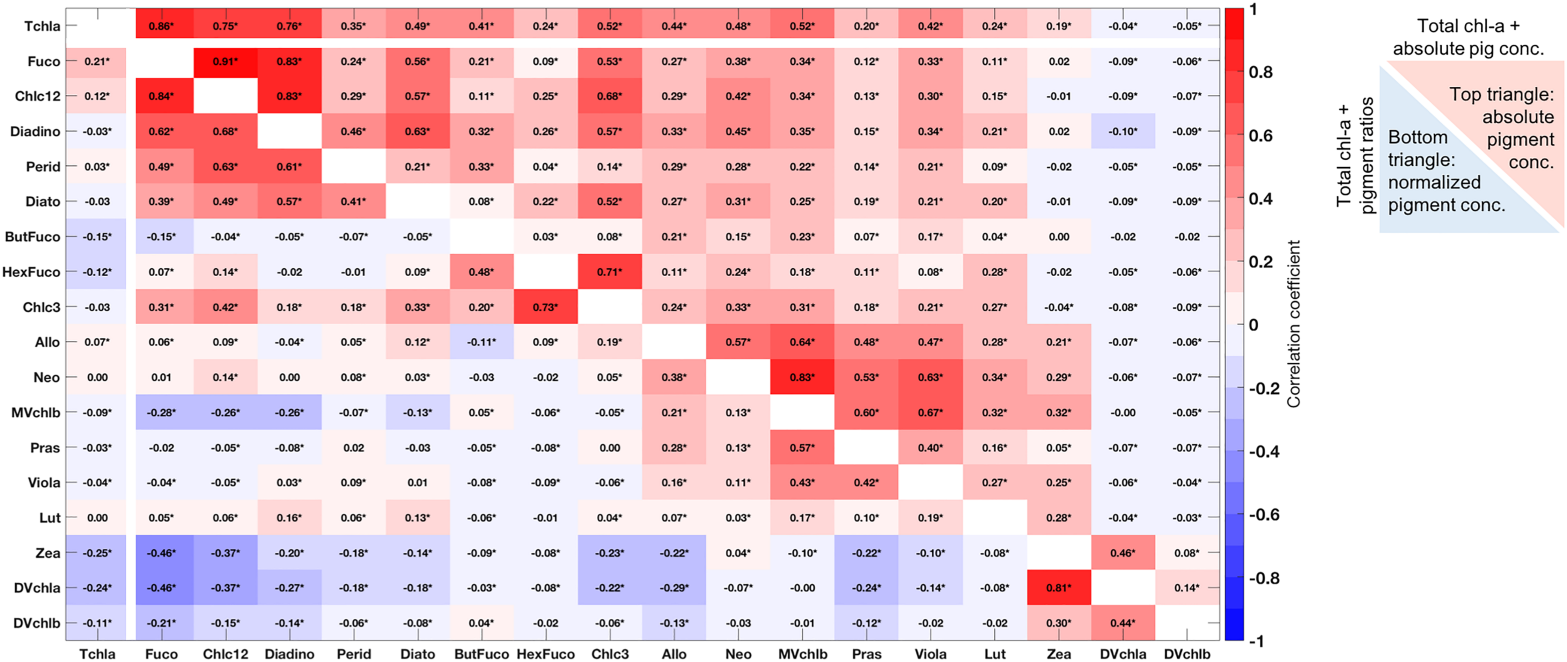
Pearson correlation coefficient (*R*) between all pigments in the global dataset: absolute concentration (upper right portion) and normalized to total chlorophyll‐*a* concentration (lower left portion). The top row shows *R* values between absolute pigment concentrations and total chlorophyll‐*a*; the far left row shows *R* values between pigment concentrations normalized to total chlorophyll‐*a* and total chlorophyll‐*a*. The warm colors indicate positive correlation; the cool colors indicate negative correlation. Stars denote significant correlations (i.e., null hypothesis rejected using Student's *t* test, *p*<0.05).

A total of 4,480 samples were used in the global dataset and 1,607 samples in the local dataset for subsequent analyses after applying the above data quality assurance procedures. The data were further quality controlled by setting all pigment values below established HPLC method detection limits to zero (Van Heukelem & Thomas, 2001). Prior to any of the following analyses, all pigments were normalized to total chlorophyll‐*a* concentration. As the following statistical and network analyses are correlation‐based, the Pearson correlation coefficients (R values) between the remaining seventeen pigments are used. Correlation coefficient values were calculated for the global dataset among these 17 pigments (both absolute concentrations and ratios to Tchla) and with Tchla (Figure 2).

### 2.3 Hierarchical Cluster Analysis

Hierarchical cluster analyses were performed separately on both the global dataset and on the local dataset for each time series observatory site using all seventeen pigments described above, normalized to Tchla. The correlation distance (1‐R, where R is the Pearson correlation coefficient between phytoplankton pigments) and Ward's linkage method (the squared inner distance), following Latasa and Bidigare (1998) and Catlett and Siegel (2018), are calculated in MATLAB (R2018a) with the “pdist” and “linkage” functions, respectively. The cophenetic correlation coefficient and *p*‐values were computed in MATLAB with the “cophenet” function for all dendrograms to evaluate the validity of the hierarchical cluster analyses performed here (Legendre & Legendre, 1998). The cophenetic correlation coefficient compares the distance matrix generated during the cluster analysis with the linkage distances determined for construction of the dendrogram. The correlation coefficient can vary from 0 to 1: values closer to one indicate high correlation between these distances, which suggests that the resulting dendrogram accurately depicts the distances between the input parameters (in this case, the pigment ratios to Tchla). The *p*‐value indicates the significance of this correlation (values <0.05 are considered significant). If these metrics suggested that the dendrogram was accurate and significantly related to the distance matrix, then the “cluster” function was used in MATLAB (R2018a) to define the linkage distance cutoff for a maximum number of taxonomically relevant clusters, using the linkages calculated using the Ward method.

### 2.4 Empirical Orthogonal Function Analysis

An EOF analysis was performed on both the global dataset and on each time series observatory dataset to further evaluate the co‐variability in groups of phytoplankton pigments (following C. R. Anderson, Siegel, et al., 2008 and Catlett & Siegel, 2018). An EOF analysis decomposes the data into dominant orthogonal functions descriptive of the major modes of variability in the dataset. The percent variance explained by each mode decreases with higher modes; that is, Mode 1 describes the most variance in the dataset and only the lowest few modes are useful for interpreting a dataset. For each mode, an EOF analysis results in both the loadings over the entire dataset and amplitude functions for each sample. The loadings describe the correlations between each mode and the input variables (in this case, pigment ratios to Tchla). The amplitude function describes the strength of each mode for each sample. The summed product of the loadings and amplitude functions over all of the EOF modes enables reconstruction of the original dataset. Pigments concentrations (normalized to Tchla) were mean‐centered and normalized by their standard deviation before EOF analysis. Correlations between the dominant global EOF modes and several relevant environmental variables (specifically latitude, temperature, salinity, annual mean nitrate concentration, and water depth from bathymetry) were also evaluated.

## 3 Results

### 3.1 Global HPLC Pigment Data

The global HPLC pigment dataset features a broad range of chlorophyll‐*a* concentrations (0.006–26 mg/m^3^) from oligotrophic to eutrophic conditions (Figure 1a). The log‐transformed chlorophyll‐*a* data follow an approximately normal distribution (Figure 1b) with a median global value of 0.31 mg/m^3^. The global temperature data (Figure 1c) and annual mean nitrate concentration (Figure 1d) show a bimodal distribution with regions of low and high temperature and nitrate concentration well represented in the dataset.

Nearly all pigments are positively correlated with Tchla (Figure 2, top row). The absolute concentration of the seventeen pigments are also nearly all positively correlated with one another (Figure 2, upper right portion of matrix), with the exception of the pigments unique to picophytoplankton (DVchla and DVchlb), which are positively correlated only with each other and with Zea (which is also found in nanophytoplankton) and not with other pigments. However, when the pigments are normalized to total chlorophyll‐*a* (Figure 2, bottom left portion of matrix), the strong positive correlations between pigment pairs are lost and the remaining significant correlations with Tchla are largely among related groups of pigments (left column of Figure 2). In the statistical analyses to follow, pigment concentrations are normalized to Tchla to maximize the strength of connections among related pigments, with the goal of separating groups of pigments detectable by existing and future global remote sensing algorithms.

### 3.2 Local HPLC Pigment Data

The six local observatory sites used in this analysis represent a broad range of geographic and ecological conditions, and thus very different median Tchla concentrations and accessory pigment ratios (Table 2). While many of the local sites have year‐round sampling, at the Palmer LTER and Bowdoin Buoy, the sampling is seasonal (local spring and summer) and thus represents fewer months of the year. The highest median Tchla concentration (3.30 mg/m^3^) is at the Bowdoin Buoy, which is in a productive estuary; the lowest median Tchla concentration (0.17 mg/m^3^) is at BOUSSOLE, which is in the Mediterranean Sea. The variations in ratios of biomarker pigments to Tchla at these different sites suggest that different phytoplankton communities dominate at these sites (Table 2). Further, some pigments were never present or always measured below instrument detection level (and thus were set to equal zero) at the local sites (Table 2), whereas the global dataset represents a wider variety of samples such that all 17 pigments in the global dataset have a median value or median ratio to Tchla above zero.

**Table 2:** Statistics for global HPLC dataset and local observatory datasets. The bold values indicate the highest (red) and lowest (blue) values for each parameter. Stars indicate that the value for a given dataset is significantly different from the median values of all other datasets (two‐way ANOVA, *p*<0.001)

Dataset	Sampling months	# samples	Dominant group from pigment ratios	Median Tchla (mg m^‐3^)	Tchla range (mg m^‐3^)	Median Fuco: Tchla	Median Perid: Tchla	Median 19but: Tchla	Median 19hex: Tchla	Median Allo: Tchla	Median MVchlb: Tchla	Median Zea: Tchla	Cophenetic correlation coefficient	Groups ID'ed in hierarchical cluster analysis	Linkage distance cutoff
Global	All	4,480	Haptophytes	0.31	0.01‐25.0	0.140	0.017	0.051	0.193	0.001	0.045	0.043	0.83, p<<0.001	**4:** Diatoms and dinos, haptos, green algae, cyanos	1.0
BOUSSOLE	All	225	Haptophytes	**0.17**	0.05‐5.3	**0.056**	0.019	**0.068**	**0.253**	0.020	**0**	0.134	0.89, p<<0.001	**5:** Diatoms, dinos, cyanos, cryptos, haptos	1.0
Bowdoin Buoy	Apr‐Oct	161	Diatoms	**3.30**	0.55‐9.1	0.275	**0.039**	**0.003**	**0.010**	0.057	**0.072**	0.008	0.92, p<<0.001	**5:** Green algae, cryptos, dinos, haptos, diatoms	1.0
CARIACO	All	81	Cyanobacteria	0.20	0.10‐8.5	0.065	0.023	0.023	0.102	**0**	0.046	0.294*	0.95, p<<0.001	**5:** Diatoms, dinos, haptos, green algae, cyanos	0.8
MVCO	All	278	Diatoms	1.95*	0.10‐9.7	**0.321**	0.025	0.006	0.031	0.027	0.068	0.017	0.93, p<<0.001	**4:** Haptos, dinos, green algae, diatoms	1.0
Palmer	January	155	Diatoms	1.28	0.11‐27.6	0.284*	**0.014**	0.011	0.136	0.182*	0.027	**0**	0.93, p<<0.001	**6:** Diatoms, green algae, prasinos, crysos + dinos, haptos, cryptos	0.95
Plumes and Blooms	All	711	Diatoms	1.70*	0.16‐28.4	0.269	0.030	0.017	0.077	0.016	0.048	0.026	0.87, p<<0.001	**5:** Green algae, diatoms, dinos, haptos, cyanos	0.8

Abbreviations: ANOVA: analysis of variance; HPLC: high‐performance liquid chromatography.

### 3.3 Global Hierarchical Cluster Analysis

**Figure 3:**
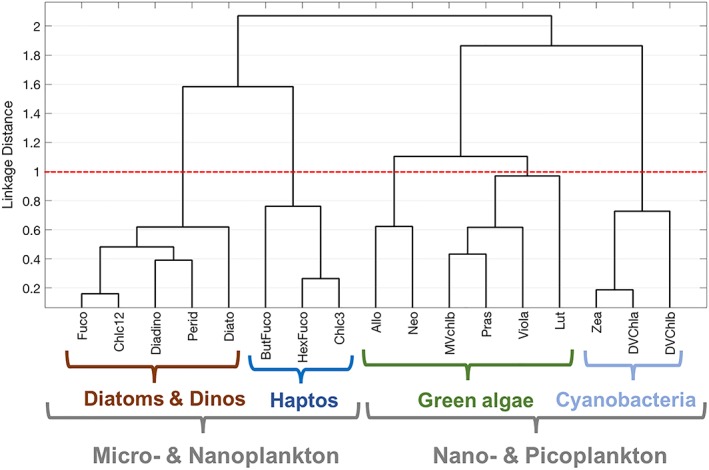
Hierarchical clustering of phytoplankton pigment ratios to total chlorophyll‐*a* for the global dataset*.* The four major pigment communities (diatoms + dinoflagellates, haptophytes, green algae, and cyanobacteria) are identified based on a linkage distance cutoff of 1.0 (red dashed line). The suggested phytoplankton cell size classes for each group are delineated with brackets.

The global hierarchical cluster analysis illustrates that there are four groups of phytoplankton pigments that dominate co‐variability of the global pigment suite, inferred from the groups of pigments clustered in each branch and the distribution of these pigments across taxonomic groups (Figure 3). The cophenetic correlation coefficient for the global dataset is high (0.83; Table 2) and the p‐value is extremely low (≪0.001), which indicate that the dendrogram is a significant and appropriate representation of the distances between pigment ratios to Tchla. Marker pigments indicate specific groups of phytoplankton (following Table 1): for the diatom and dinoflagellate group, the strong association between Fuco and Perid, respectively; for haptophytes, HexFuco and Chlc3; for green algae, the combination of MVchlb with Pras and other accessory carotenoid pigments; and for cyanobacteria, the presence of DVchla and Zea. While some of these pigments are shared between groups (i.e., Chlc12 are found in diatoms and dinoflagellates, but also in haptophytes; Table 1), the grouping here reflects the strength of the correlation coefficient between pigments normalized to Tchla (so Chlc12 is here most strongly correlated with other pigments most commonly found in diatoms and dinoflagellates; Figure 2). The linkage distance cutoff for these four taxonomic groups was 1.0 (Table 2).

The dominant groups can also be described in terms of their contributions to the three major size classes of phytoplankton (picophytoplankton, nanophytoplankton, and microphytoplankton; Figure 3). Here, the haptophytes, which are nanophytoplankton, cluster more closely with other red algae (the microsized to nanosized phytoplankton), while the green algal group (also nanophytoplankton) clusters more closely with the picophytoplankton. Finally, cryptophytes (which are nanosized to picosized red algae that uniquely contain alloxanthin) cluster or group with the nanosized green algal community across all analyses presented here, but are not as strongly correlated with the pigments in this group.

### 3.4 Global EOF Analysis

**Figure 4:**
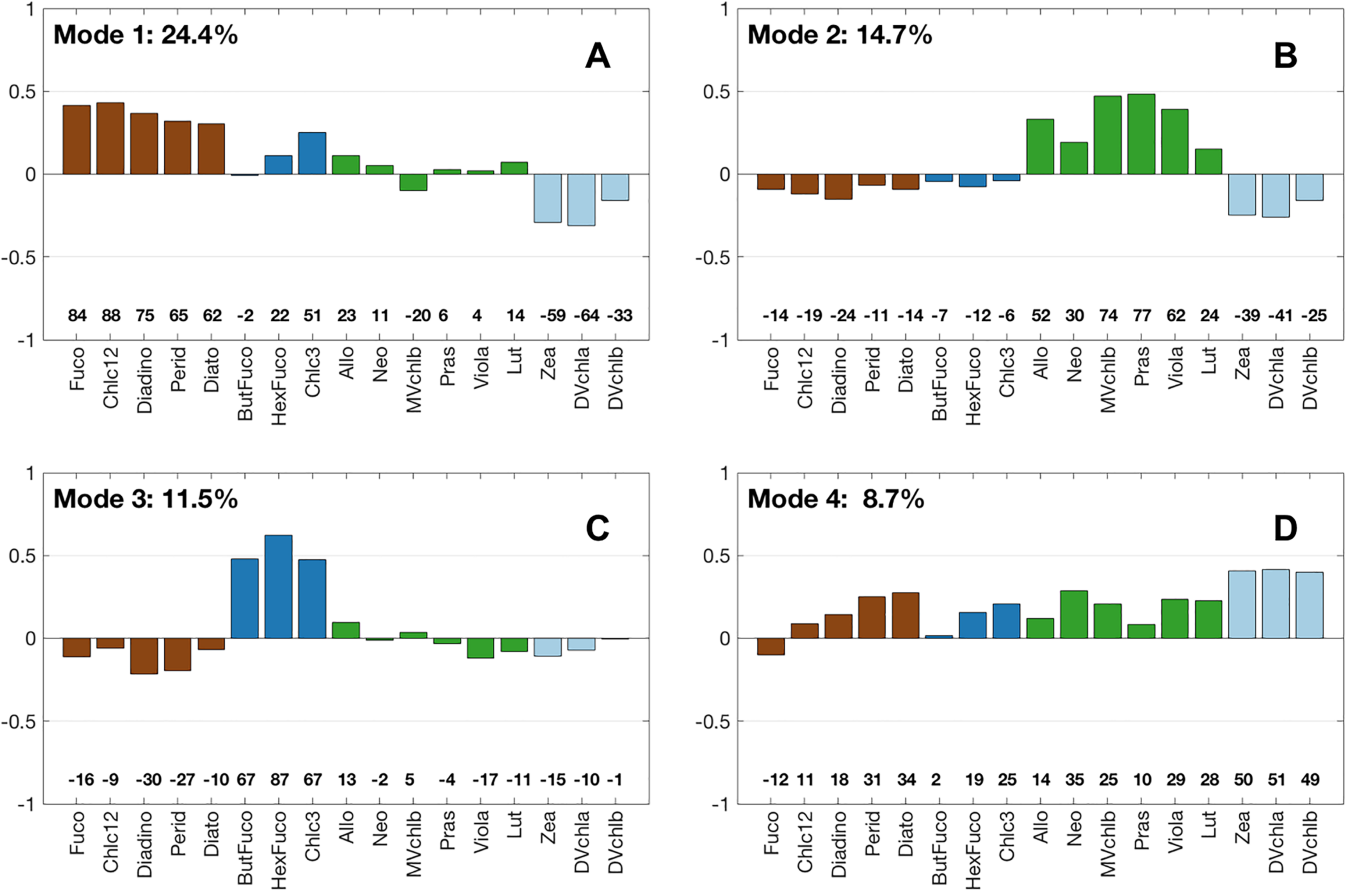
Loadings for empirical orthogonal function modes (a) 1, (b) 2, (c) 3, and (d) 4 for the global dataset. The mode number and percent variance explained by that mode are listed above each plot. Numbers above each pigment represent the correlation coefficient of that pigment with the given mode multiplied by 100. Pigments are colored by major taxonomic group: cyanobacteria (light blue), haptophytes (dark blue), diatoms, and dinoflagellates (brown), green algae (green).

**Figure 5:**
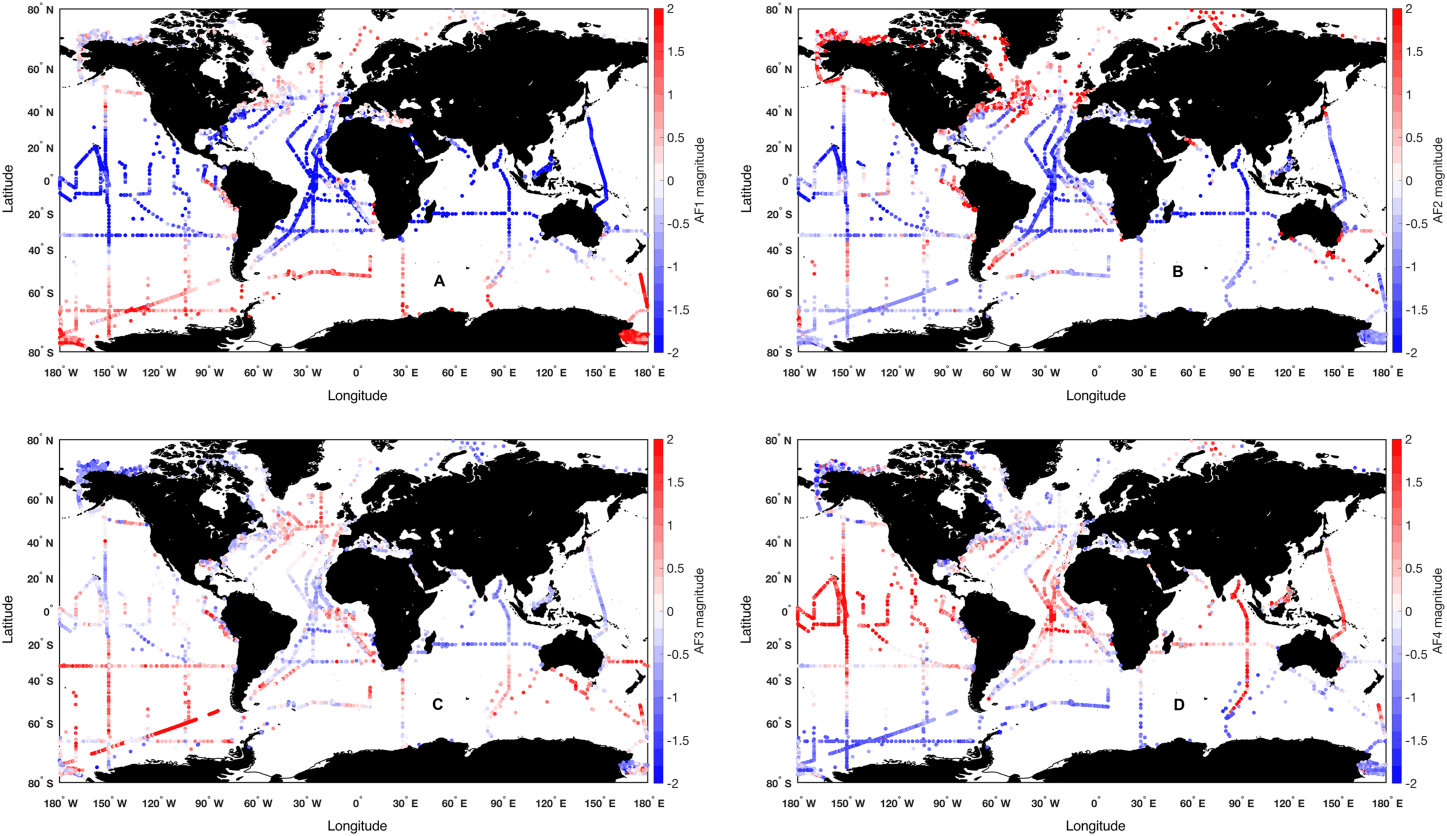
Spatial distribution of amplitude functions (AFs) for empirical orthogonal function (EOF) modes (a) 1, (b) 2, (c) 3, and (d) 4 for the global dataset. Positive values are red, negative values are blue.

The dominant modes of the global EOF analysis are represented by a set of loadings showing the relative contribution of each pigment ratio to each mode (Figures 4 and S2), as well as an amplitude function that shows the contribution of each mode to the covariability of the pigment suite spatially (Figure 5). The Pearson correlation coefficients (*R* values) between EOF loadings and pigment ratios to total chlorophyll‐*a* are also presented (Figures 4 and S2). The first six modes describe 72% of the variance in the dataset. However, only the first four modes are examined here, as the fifth and sixth modes have weak taxonomic associations (Figure S2).

EOF Mode 1 (Figure 4a) accounts for nearly one quarter of the variability in the dataset (24.4%) and can be interpreted as a diatom‐ and dinoflagellate‐dominated community when the mode's amplitude function is positive and a picophytoplankton‐dominated community when it is negative. The pigments associated with diatoms and dinoflagellates are most strongly positively correlated with Mode 1. The pigments associated with cyanobacteria and picophytoplankton are strongly negatively correlated with Mode 1. Neither haptophyte nor green algal pigments make substantive contributions to the loadings of Mode 1. Mode 1 shows spatial patterns that are negative (cyanobacteria) at low latitudes and positive (diatoms and dinoflagellates) at high latitudes consistent with this interpretation (Figure 5a).

EOF Mode 2 (Figure 4b) explains 14.7% of the variance in the dataset and is strongly positively correlated with pigments related to the green algal communities, including prasinophytes (which uniquely contain Pras). Mode 2 is negatively and moderately correlated with all other groups: diatoms and dinoflagellates, haptophytes, most strongly with cyanobacteria pigments. When this mode's amplitude function is positive, it explains a dominance of green algae in the phytoplankton community, with strongly positive samples found near the coasts (Figure 5b).

EOF Mode 3 (Figure 4c) explains 11.5% of the variance and is strongly positively correlated with pigments found in haptophytes, particularly HexFuco which is found in both coccolithophores (i.e., *Emiliania huxleyi*) and *Phaeocystis* spp (Table 1). This mode is negatively correlated with all other groups, particularly with the diatom and dinoflagellate cluster of pigments. Mode 3 explains a dominance of haptophytes when the amplitude function is positive and is found at mid latitudes, as a transition between the low‐ and high‐latitude phytoplankton communities (Figure 5c).

Finally, EOF Mode 4 (Figure 4d), which explains 8.7% of the total variance, is positively correlated with nearly every pigment, notably DVchla, DVchlb, and Zea, which are markers for cyanobacteria. The only pigment that is negatively correlated with Mode 4 is Fuco. While this correlation is low (*R* = −0.12), this result suggests that Mode 4 can in principle partition diatoms from the other groups. When the Mode 4 amplitude function is positive, a mixed assemblage is present with an emphasis on the cyanobacteria community at low latitudes, while samples with negative amplitude function values are found at high latitudes (Figure 5d).

Few environmental variables were either positively or negatively correlated with the first four EOF amplitude functions. The amplitude function for the first mode is slightly negatively correlated with temperature (*R* ^2^=0.36) and positively correlated with nitrate concentration (*R* ^2^=0.36), but the amplitude functions for all other modes are uncorrelated with temperature, salinity, nitrate concentration, or water depth (*R* ^2^≤0.17). The role of data source as a dummy variable was also examined to determine whether the dominant modes of variability in the dataset were correlated with the lab where the HPLC pigment data were processed; none of the modes identified by the EOF analysis were correlated with the source of the data (*R* ^2^≤0.14).

### 3.5 Local Hierarchical Cluster and EOF Analyses

**Figure 6:**
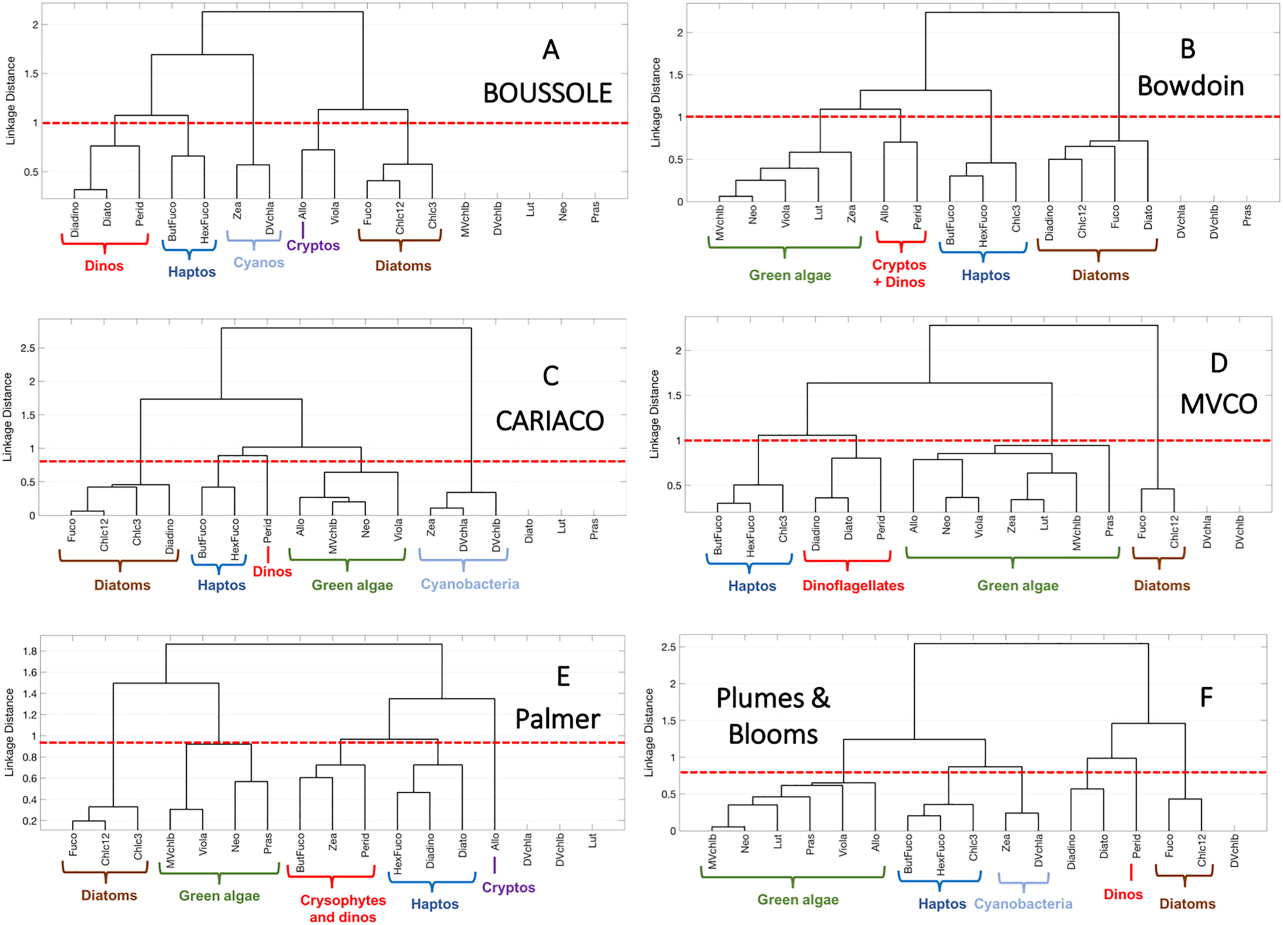
Hierarchical clustering of phytoplankton pigment ratios to total chlorophyll‐*a* at six observatory time series sites: (a) BOUSSOLE, (b) Bowdoin, (c) CARIACO, (d) MVCO, (e) Palmer LTER, and (f) Plumes and Blooms. The major pigment‐based communities are delineated with brackets. Pigments that were not measured or measured below detection 75+% of the time were not included in the cluster analysis, but are listed on the *x* axis. The red lines indicate the linkage distance cutoff for taxonomically relevant groups (Table 2).

Hierarchical cluster and EOF analyses for each time series observatory site in the local dataset show clear differences from the global scale results (Figures 6 and S3). On global scales, four phytoplankton groups could be distinctly identified from both hierarchical clustering and EOFs, but on local scales, more and different phytoplankton groups emerge. The cophenetic correlation coefficients for the local datasets range between 0.87 and 0.95 and the *p*‐values are all extremely low (≪0.001), which indicates that the distance matrices for pigment ratios to Tchla are accurately represented by the dendrograms for all sites. Between four and six taxonomically relevant groups are then identified at each site, with linkage distance cutoffs between 0.8 and 1.0 (Table 2). The differences between observatory sites, and between the local and global data, are considered here.

There are four phytoplankton groups that are clearly separated by hierarchical clustering at MVCO and the Bowdoin Buoy, five groups identified at BOUSSOLE, CARIACO, and Plumes and Blooms, and six groups identified at the Palmer LTER (Table 2). Both global and local hierarchical cluster analyses identify distinct groups of cyanobacteria, haptophytes, and green algae (Figures 3 and 6). However, some of the groups identified in the global dataset do not emerge at some local sites. For instance, cyanobacterial pigments are not found at MVCO, Palmer, or the Bowdoin Buoy. Conversely, new groups emerge on local scales that were not identified on global scales. Notably, dinoflagellate biomarker pigments (Perid and others; Table 1) separate from diatom pigments (Fuco and others) at all six sites (Figure 6). In the global cluster analysis, dinoflagellate pigments group with diatom pigments and thus the dinoflagellates are indistinguishable as a separate taxonomic group; at all sites except Plumes and Blooms, Perid is quite distant from Fuco. Additionally, the cryptophyte biomarker pigment (Allo) separates from other red algal pigments at BOUSSOLE and Palmer, and clusters with dinoflagellate pigments at the Bowdoin Buoy. In the global analysis, Allo clusters with green algal pigments, suggesting the co‐occurrence of cryptophytes and green algae when viewed on global scales. Finally, at the Palmer LTER, Perid groups with crysophytes based on the affiliation of ButFuco and Zea; in the global dataset and at other observatory sites, these pigments cluster with haptophytes and cyanobacteria, respectively. While the new groups identified on local scales are different than the groups found in the global hierarchical cluster analysis, all of these groups can still be identified by diagnostic biomarker pigments.

The local EOF analyses (Figure S3) show similar results to the local hierarchical cluster analyses. The same major taxonomic groups are identified in the EOF analyses as are identified in the hierarchical cluster analyses for each site; again, the local results show more and different groups emerging in the EOFs than the global results. On global scales, dinoflagellate pigments separate from diatom pigments only in Mode 4 (Figure 4), but the pigment loading for Perid is not highly correlated with Mode 4 (*R*=0.31). Dinoflagellate pigments separate from all other pigments in at least one mode from Modes 2 to 4 for each observatory site (Figure S3) and Perid is highly correlated with the mode in which dinoflagellates are best represented (*R*=0.47–0.79). At Plumes and Blooms, a pigment cluster emerges of photoprotective pigments Diadino and Diato (Figure 6): these pigments are found in all red algae and some green algae (Table 1). The EOF analysis shows that these pigments are positively correlated with Mode 2 (haptophytes) and Mode 3 (dinoflagellates) and negatively correlated with Mode 4 (diatom pigments). Thus, they are not associated with one cluster of pigments but form their own, highly linked cluster.

As the local results represent time series sampling while the global results are individual points in time, the EOF results for the local analyses often show groups of phytoplankton co‐occurring in different modes, capturing different seasons of sampling. For instance, at MVCO, which is sampled year‐round, dinoflagellate and haptophyte pigments are both positively correlated with Mode 2, while dinoflagellates separate from all other groups in Mode 3, and then dinoflagellate and green algal pigments are both negative correlated with Mode 4 while haptophyte pigments are positive correlated with Mode 4 (Figure S3d). In this case, dinoflagellates can be separated from other groups in 3 of the first 4 modes, but each mode offers new ecological information for further interpretation over a seasonal cycle.

## 4 Discussion

The statistical methods applied to global and local surface ocean HPLC pigment observations allow us to characterize four robust taxonomic groups of phytoplankton on global scales, and more and different groups of phytoplankton on local scales. Here, the dominant information content in HPLC pigments across varying spatial scales is discussed. The construction of the global and local surface ocean HPLC datasets and the selection of statistical methods are considered, as the information content in HPLC pigments is weakened without quality control. Finally, in light of the results found here, suggestions are made for using HPLC data in global and local satellite algorithm development and calibration, including the utility of employing biomarker pigment concentrations to denote the main phytoplankton communities identified here. Our results suggest that robust communities of phytoplankton can be identified on varying spatial scales, but the limitations of the HPLC pigment dataset used will necessarily limit the phytoplankton communities obtained and the satellite algorithms that can be created.

### 4.1 Evaluating the Dominant Information Content in HPLC Pigments Across Varying Spatial Scales

Pigment‐based methods remain some of the most common ways to assess phytoplankton community structure across taxonomic groups, despite any associated limitations. While phytoplankton diversity is vastly more complex than the results presented here might suggest [i.e., de Vargas et al., 2015], HPLC data are available on global scales, across biogeographic provinces, seasons, and environmental conditions, and at time series observatory sites to track long‐term changes in pigment composition and concentration. A goal of this analysis is to determine the maximum amount of information that can be determined about global vs. local phytoplankton community structure from HPLC pigments with application to remote sensing algorithm calibration and validation. The results presented here demonstrate that the relationships between and among groups of phytoplankton pigments can reliably be used to describe four distinct taxonomic groups of phytoplankton on global scales: diatoms and dinoflagellates, cyanobacteria, green algae, and haptophytes. On local scales, up to six taxonomic groups can be successfully separated from HPLC pigments, but the groups that emerge vary based on the dominant taxa at each observatory site.

Globally, proportion of samples with high concentrations of dinoflagellate (Perid) and crypotophyte (Allo) biomarker pigments are rare enough in the global dataset that these groups are not independently identified by the statistical methods applied in the global analyses. However, on local scales, HPLC pigments often provide higher taxonomic resolution about the phytoplankton community. The hierarchical cluster (Figure 6) and EOF analyses (Figure S3) of each local dataset identify more and different phytoplankton groups than were detected in the global dataset (Figures 3 and 4). Notably, dinoflagellate (Perid) pigments separate from diatom (Fuco) pigments at every site in the local dataset, but dinoflagellate and diatom pigments cluster together in the global dataset. Cryptophyte (Allo) pigments and crysophyte (ButFuco and Zea) pigments also cluster individually from other red algal pigments in the local data, but these pigments generally cluster with either the red algal or haptophyte pigments in the global dataset. The information content of local scale HPLC pigment data provides higher taxonomic resolution than the global dataset, as more groups can be identified at most of the observatory sites than on global scales. Importantly, HPLC pigments allow for the identification of dinoflagellates on local scales, which is also relevant to regional ecology, fisheries, and human health, as many dinoflagellate species can form harmful algal blooms (e.g., D. M. Anderson, Burkholder, et al., 2008). HPLC pigments measured at time series sites offer a larger dynamic range of pigment concentrations sampled over the course of a seasonal cycle that captures seasonal successional patterns of phytoplankton groups, rather than the global dataset which encapsulates the entire global range of possible combinations of pigments.

The results presented here demonstrate the potential and limitations of using HPLC pigment ratios to develop global and local remote sensing algorithms. While some methods purport to identify as many as eight distinct phytoplankton groups from HPLC pigments (e.g., CHEMTAX; Mackey et al., 1996), this analysis suggests that only four and up to six groups can be identified from pigments, even from high resolution local‐scale sampling. Given the shared pigments between many phytoplankton groups (Table 1), the cluster and EOF analyses allow for differentiation between groups, but to a point. As the groups that can be reliably identified from pigments on local scales are different than the groups that can be identified on global scales, HPLC pigments can be used on local scales to create and validate remote sensing algorithms that target local, pigment‐specific phytoplankton groups (such as dinoflagellates). Understanding the differences in phytoplankton taxonomic resolution on varying spatial scales is crucial to constructing applicable and relevant satellite remote sensing models for the present and future ocean (Bracher et al., 2017 and references therein).

### 4.2 Considerations in Synthesizing and Analyzing a Global Surface Ocean Phytoplankton Pigment Dataset

In order to evaluate the suitability of HPLC pigments for distinguishing between phytoplankton group across varying spatial scales, consistent data are required, with spurious samples removed and inconsistent or redundant data sources eliminated before analysis. Thus, in this case, more data are not necessarily better. Rather, two distinct, coherent datasets of global and local scale samples, with clear criteria for inclusion, were essential. The careful inclusion of these datasets allows for the associations between and among HPLC pigments to be investigated with as few spurious samples included as possible. Here, the choices required and challenges involved in curating and analyzing such a data synthesis are discussed.

In constructing a global dataset of HPLC samples with contributions from over sixty distinct oceanographic cruises and sampling programs, there are bound to be sources of uncertainty and caveats to the conclusions presented here. While community‐defined recommendations for best practices exist at all stages of analysis for sampling seawater, filtering seawater, storing filters before analysis, and for the analysis itself (i.e., https://oceancolor.gsfc.nasa.gov/docs/technical/), it would be impossible to ensure that these protocols were followed for every sample in this dataset. Thus, while all efforts have been made to remove spurious data from the global assemblage (see *Quality control and quality assurance*, above), some sources of error may remain. However, other sources of potential uncertainty in this analysis can be quantified and are described in further detail.

The role of data source was considered carefully throughout this analysis. The EOF amplitude functions for the global dataset were not strongly correlated with any one data source (*R* ^2^≤0.14). When the mean values of several biomarker pigments are compared for each data source, it is clear that the sample collection for some data sources was biased to specific geographic regions (Figure S1 and Table S2). The samples from the DiTullio lab are overwhelmingly from the Peruvian Upwelling Zone and the Southern Ocean (Figure S5), regions dominated by diatoms and haptophytes. Unsurprisingly, the mean values of Fuco and HexFuco are significantly higher than the mean values for other analytical facilities (Table S2). Similarly, the AWI samples were all taken from low to mid latitudes, concentrated in the equatorial Atlantic and Pacific Oceans (Figure S5); these regions are dominated by cyanobacteria, which is reflected by the significantly high mean concentration of Zea for this analysis facility. The local dataset includes six time series observatory sites: naturally, the data from each of these sites has high geographic variation and very different biomarker pigment concentrations and ratios (Table 2)—with the exceptions of the Bowdoin Buoy and MVCO, which are geographically close but with different phytoplankton communities (e.g., cryptophytes group with dinoflagellates at the Bowdoin Buoy but with green algae at MVCO).

The construction of a large HPLC pigment dataset across multiple sources includes the decision to require a minimum phytoplankton pigment suite and to average data over space, depth, and/or time; these decisions may lead to differences in the conclusions of pursuant statistical analyses. Comparable global analyses of HPLC pigment data have grouped samples by season [i.e., Swan et al., 2016] or integrated pigment values over the euphotic zone [i.e., Uitz et al., 2006] prior to analysis. As our goal was assessing information content in surface ocean HPLC pigment observations for remote sensing applications, the quality of the global and local datasets (including the depth of sampling, consistency of the pigments measured, and the geographic distribution of samples) was central to our conclusions. Strict criteria were used to construct the dataset used for this analysis. A minimum number of pigments were required to be measured for inclusion in the dataset, samples were processed at a limited number of analytical facilities, and were not averaged over space or depth. These criteria necessarily excluded some datasets from inclusion.

Similarly, the selection of statistical methods was carefully considered in this analysis. The co‐variability observed between pigments and pigment ratios in the global dataset (Figure 2) creates difficulties for statistical methods that model phytoplankton groups from observations of HPLC pigments. Some common methods, such as the DPA, do not make assumptions about co‐linearity in the pigment data that would be complicated by the observed co‐variability; however, other methods rely on assumptions of linear contributions between accessory pigments or between accessory pigments and Tchla. For instance, CHEMTAX is a widely used method (Mackey et al., 1996) that aims to estimate several phytoplankton groups from HPLC pigments based on assumptions of their contributions to Tchla. CHEMTAX assumes that individual pigments or combinations of pigments correspond to unique groups of phytoplankton, allowing for statistical separation of phytoplankton group contributions to Tchla, and that the contributions of individual phytoplankton pigments to each taxonomic class are known. On global scales, taxa‐specific pigment ratios are not expected to be constant. Even on local scales, where pigment contributions can be better defined and constrained for taxa of interest, direct comparisons between CHEMTAX and other methods of phytoplankton identification are often inconsistent (e.g., Havskum et al., 2004; Kramer et al., 2018; Pan et al., 2011). Finally, CHEMTAX assumes linear independence between the pigments, which is inconsistent with the data compiled here (Figure 2). Multicollinearity dilutes the significance of individual pigments in the matrix inversion due to the correlation between pigments (Legendre & Legendre, 1998). As several of the underlying assumptions of CHEMTAX are not supported by the global dataset, it was not used here.

The data‐driven methods presented here do not require a priori assumptions to determine group membership, but rather rely on the similarity in pigment composition and concentration between groups of samples to define taxonomic phytoplankton communities across spatial scales. Similarly, only the ratios of individual pigments to Tchla are used here to reduce the between‐group correlations of nearly all phytoplankton pigments. The global data did not support an attempt to further parse the main communities detected here into more distinct groups. Thus, differences are not discernable on global scales between, for example, distinct haptophyte communities, between cryptophytes and other red algae, or between prasinophytes and other green algae.

### 4.3 Potential and Limitations of HPLC Pigments for Calibration and Validation of Remote Sensing Algorithms

The results shown here demonstrate both the potential and the limitations of HPLC pigments to identify phytoplankton groups on varying spatial scales from consistent datasets. Phytoplankton pigments are a proxy for community composition that do not necessitate the human effort required for microscopic identification or for classification and validation of quantitative cell imaging [i.e., Sosik & Olson, 2007; Lombard et al., 2019]. Despite the relatively high cost and longer processing time of HPLC samples, HPLC remains a cheap, fast, and standardized method compared with high‐throughput molecular sequencing techniques [i.e., de Vargas et al., 2015; Hugerth & Andersson, 2017]. Finally, the connections between phytoplankton pigments and phytoplankton absorption allow the attribution of spectral features in both phytoplankton absorption and remote sensing reflectance to specific phytoplankton pigments [i.e., Roesler & Perry, 1995; Uitz et al., 2015; Chase et al., 2017; Catlett & Siegel, 2018; etc.], which can then be ascribed to certain taxonomic groups, as shown here.

Future satellite‐based PFT quantification will likely require hyperspectral resolution for accurate estimates of pigment concentrations that can then be used to identify distinct phytoplankton groups (e.g., Werdell et al., 2019). Hyperspectral resolution is required due to the overlap in phytoplankton pigment absorption peaks. In anticipation of these hyperspectral data, algorithms have been proposed to identify phytoplankton groups from high resolution reflectance measurements [i.e., Uitz et al., 2015; Chase et al., 2017, etc.]. On global scales, the present global HPLC pigment dataset can then be applied to develop and calibrate remote sensing algorithms that would detect up to the same four phytoplankton groups identified by the statistical methods used here. On local scales, the HPLC samples measured at each time series site could be used to calibrate and validate regional scale remote sensing algorithms that would identify more and different phytoplankton groups than the global algorithms, or that distinguished specific phytoplankton groups of interest at a local site (such as dinoflagellates, which can form toxic algal blooms).

Previous methods to detect phytoplankton groups from HPLC pigments for remote sensing algorithm validation purposes have proposed the selection of biomarker pigments to represent taxonomic groups (e.g., Uitz et al., 2006; Catlett & Siegel, 2018; etc.). The groups identified in this analysis on both global and local scales can be represented by individual pigments to serve as similar function: Fuco (globally: diatoms and dinoflagellates; locally: diatoms), HexFuco (haptophytes), MVchlb (green algae), DVchla or Zea (cyanobacteria). These pigments are meaningful for the broad taxonomic groups they represent (Table 1) and consistent with existing observations of HPLC pigments and optical oceanographic data, such as phytoplankton absorption spectra [i.e., Chase et al., 2013; Catlett & Siegel, 2018]. The local datasets used here suggest that local scale remote sensing algorithms may be able to achieve more taxonomic resolution to separate bloom species, such as dinoflagellates, from other phytoplankton using Perid. However, on global scales, future and existing satellite methods that are validated with this HPLC pigment dataset could not robustly achieve higher taxonomic resolution than the four distinct groups identified here.

The dataset used to construct or validate a remote sensing algorithm will necessarily limit the potential and applications of a remote sensing algorithm. A model developed with the global dataset used here would only be able to detect a maximum of four phytoplankton groups in the surface ocean; on local scales, the model results would not accurately reflect the ecology of that region. For instance, an algorithm for dinoflagellates cannot be built from the current global dataset. If a global remote sensing algorithm validated with the present global HPLC pigment dataset was applied to remote sensing data for a coastal region, it would likely be unable to distinguish between diatoms and dinoflagellates. A global scale algorithm would be limited to identify only the four groups that emerge on a global scale from this dataset. Thus, a global algorithm created with this dataset should only be applied on a regional or local scale with full understanding of these limitations, as some major local‐scale groups will not be able to be identified with a global‐scale algorithm constructed from this dataset. Similarly, remote sensing algorithms developed using data from one of the time series observatory sites shown here would not be suitable for global application. Many of the local sites are missing groups that appear on global scales (i.e., cyanobacteria are globally important, but their biomarker pigments are not detected at the Bowdoin Buoy, MVCO, or Palmer).

Thus, the selection of an appropriate remote sensing algorithm for the desired spatiotemporal scale of analysis is essential. Criteria will need to be established for the spatial scales where a global algorithm transitions to a local one. For example, if a global algorithm is applied and one of the four groups is missing (from an absence of the associated biomarker pigment), that missing group might provide clues of how to switch from a global to local scale algorithm. For instance, in several of the local datasets, picoplankton and cyanobacteria biomarker pigments are missing (Figure 6). Continued local and global in situ monitoring of phytoplankton communities will also be critical for determining the times and regions in which global vs. local remote sensing algorithms would be more suitable. Finally, the datasets used here are only relevant for calibration and validation of remote sensing algorithms describing conditions up to present day. These models will be limited to detect future change. Climate change is expected to alter global patterns in nutrient availability and surface ocean stratification, which may lead to increases in dinoflagellates in the global ocean [i.e., Falkowski & Oliver, 2007]. However, a model developed using the global dataset presented here would only be able to detect a mixed group of diatoms and dinoflagellates on global scales, and not a separate dinoflagellate community.

Pigment‐based methods will remain essential for building global satellite algorithms to determine phytoplankton community structure from space given the widespread availability of HPLC pigment data on varying spatial scales and over time. While there is inherent value in understanding the biogeographic distribution of phytoplankton species, ultimately many of these algorithms aim to link surface ocean biology to the downward flux of organic carbon to the deep ocean, which has implications for global climate (e.g., Guidi et al., 2016). Like many methods of phytoplankton identification, pigments do not measure biomass nor productivity nor rates of organic matter export. In order to better quantify these terms, pigment‐based methods will have to be merged with other methods that can quantify cellular carbon (e.g., flow cytometry) and describe the fraction carbon contributed by each taxonomic group. The limitations of pigment‐based methods aside, this analysis offers metrics and datasets to strengthen both existing and future remote sensing algorithms and subsequent models that will benefit from characterizing surface ocean phytoplankton community structure.

## Supporting information

Supporting Information S1
