# Supplementary material for: How Can Phytoplankton Pigments Be Best Used to Characterize Surface Ocean Phytoplankton Groups for Ocean Color Remote Sensing Algorithms?
Source: J Geophys Res Oceans. 2019 Nov 11;124(11):7557–74. doi: 10.1029/2019JC015604 (PMC7043335; doi:10.1029/2019JC015604)
Supplement: Supplementary file 1 — Supporting Information S1 [file JGRC-124-7557-s001.pdf]

Journal of Geophysical Research: Oceans

Supporting Information for

How can phytoplankton pigments be best used to characterize surface ocean phytoplankton groups for ocean color remote sensing algorithms?

Sasha J. Kramer[1,2] and David A. Siegel[2,3]

[1]Interdepartmental Graduate Program in Marine Science, University of California Santa Barbara, Santa Barbara CA, USA

[2]Earth Research Institute, University of California Santa Barbara, Santa Barbara CA, USA

[3]Department of Geography, University of California Santa Barbara, Santa Barbara CA, USA

Contents of this file

Figures S1 to S3
Tables S1 to S2

Introduction

The files here include Supplementary Figures to the main text, giving more context to the statistical analyses and dataset construction described in the manuscript. There is also a supporting table that describes the dataset in depth with associated references. This table is uploaded as a separate file. The data used in this analysis are referenced in the main text and are available at https://doi.pangaea.de/10.1594/PANGAEA.905883.

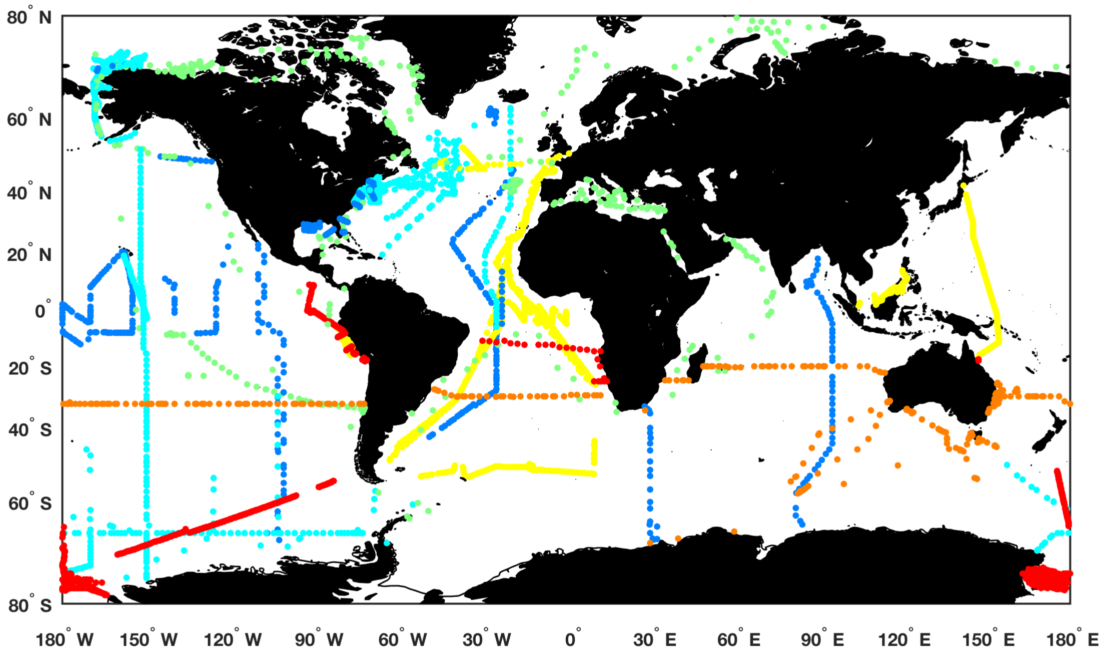

Figure S1. HPLC data analysis sources for each sample in this analysis (blue = Horn Point Labs, cyan = NASA Goddard Space Flight Center, green = Laboratoire d'Océanographie de Villefranche-sur-Mer, yellow = Alfred Wegner Institute, orange = Commonwealth Scientific and Industrial Research Organisation, red = DiTullio lab (College of Charleston).

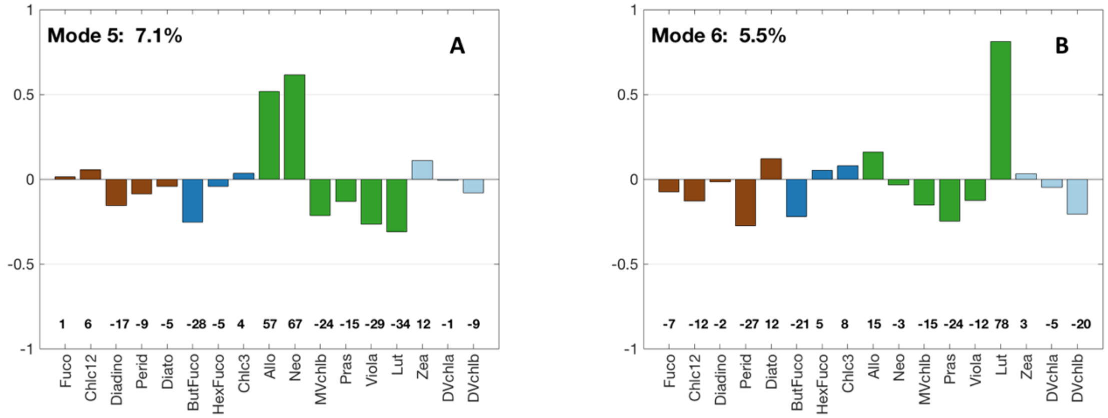

Figure S2. EOF loadings of modes (A) 5 and (B) 6 for the global dataset. The mode number and percent variance explained by that mode are listed above each plot. Numbers above each pigment represent the correlation coefficient of that pigment with the given mode multiplied

by 100. Pigments are colored by major taxonomic group: cyanobacteria (light blue), haptophytes (dark blue), diatoms and dinoflagellates (brown), green algae (green).

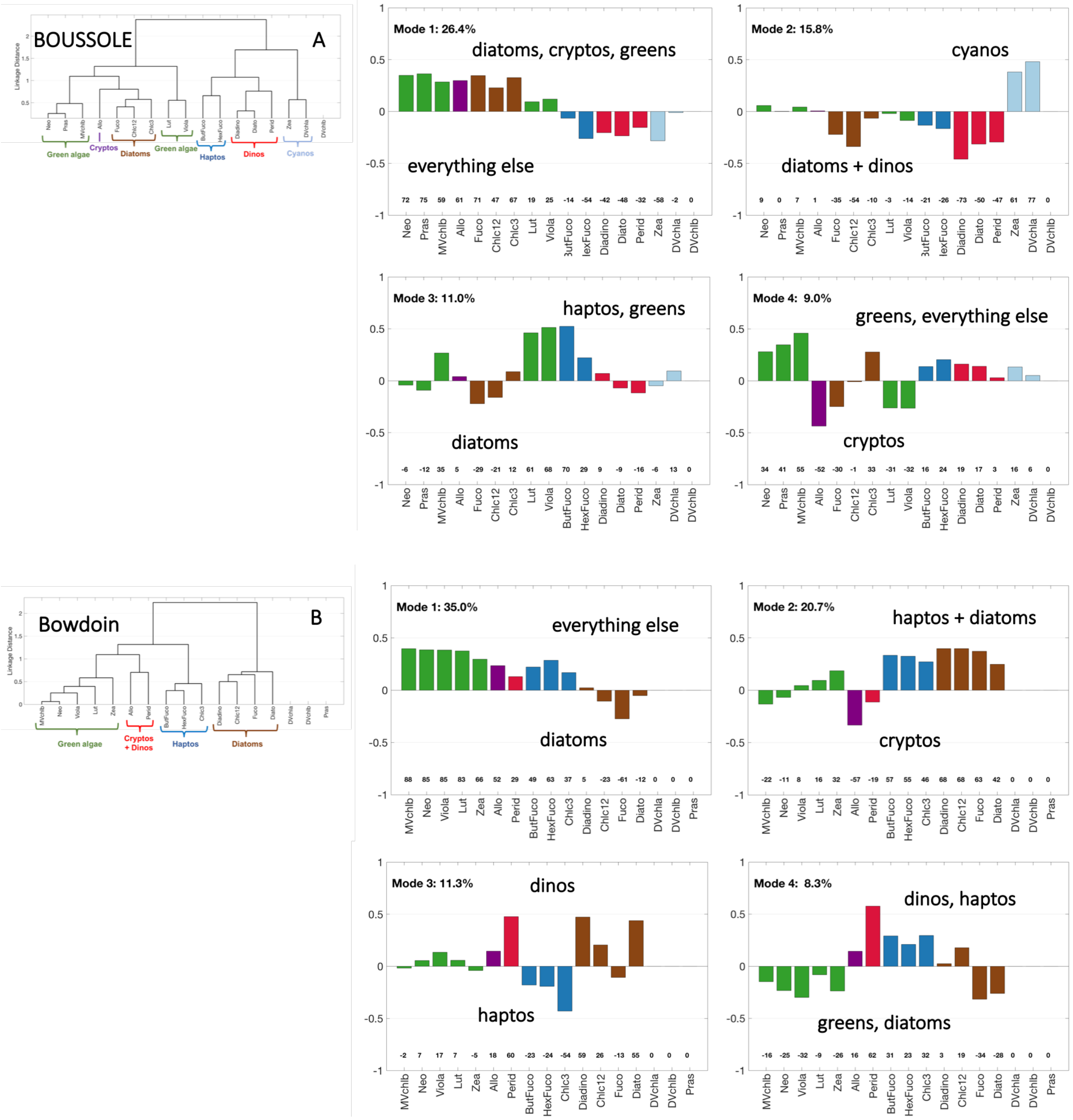

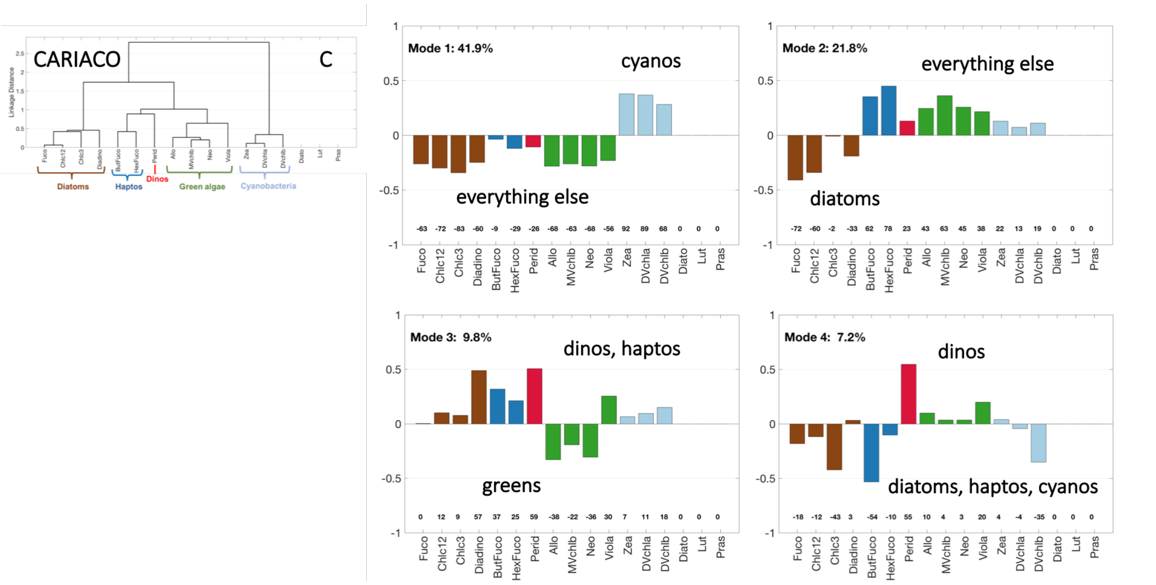
CARIACO
C
Linkage Distance
Diatoms
Haptos
Dinos
Green algae
Cyanobacteria
Mode 1: 41.9%
cyanos
everything else
Mode 2: 21.8%
everything else
diatoms
Mode 3: 9.8%
dinos, haptos
greens
Mode 4: 7.2%
dinos
diatoms, haptos, cyanos
Fuco
Chlc12
Chlc3
Diadino
ButFuco
HexFuco
Perid
Allo
MVchlb
Neo
Viola
Zea
DVchla
DVchlb
Diato
Lut
Pras

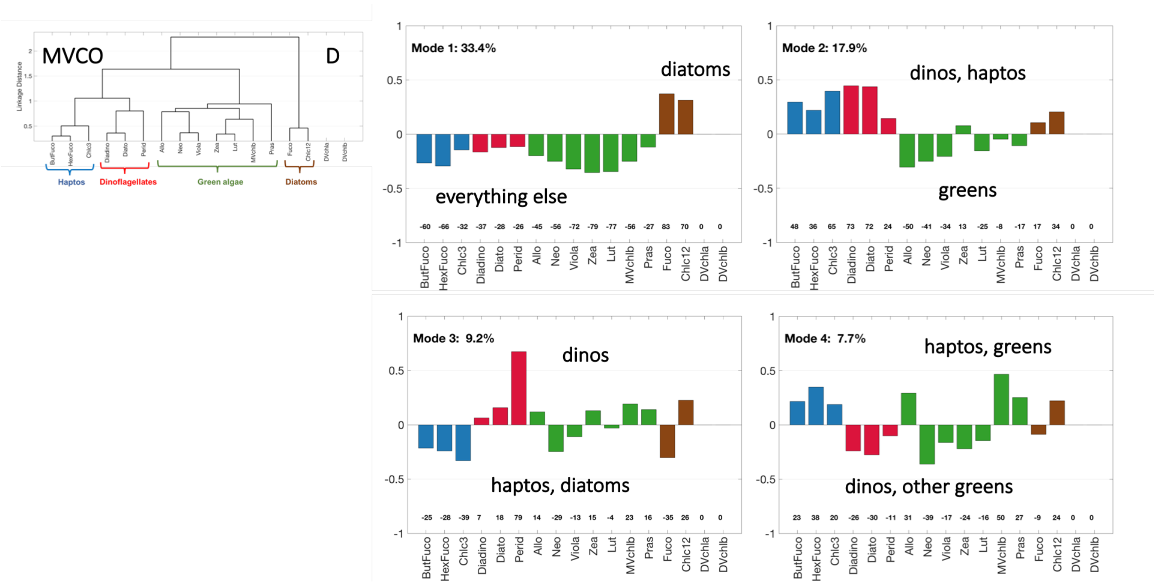
MVCO
D
Linkage Distance
Haptos
Dinoflagellates
Green algae
Diatoms
Mode 1: 33.4%
diatoms
everything else
Mode 2: 17.9%
dinos, haptos
greens
Mode 3: 9.2%
dinos
haptos, diatoms
Mode 4: 7.7%
haptos, greens
dinos, other greens
ButFuco
HexFuco
Chlc3
Diadino
Diato
Perid
Allo
Neo
Viola
Zea
Lut
MVchlb
Pras
Fuco
Chlc12
DVchla
DVchlb

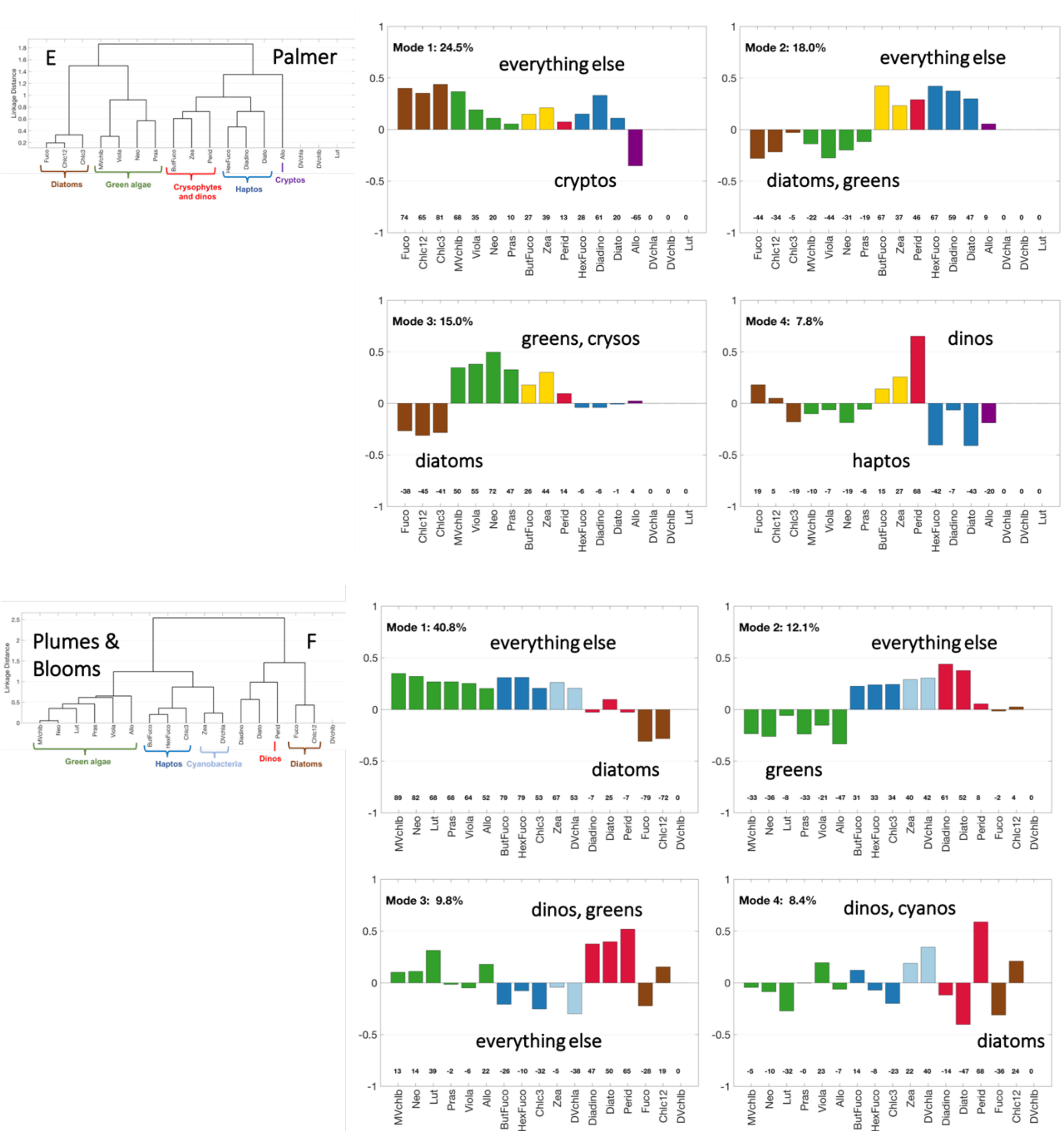

Figure S3. EOF loadings for Modes 1-4 of each observatory: (A) BOUSSOLE, (B) Bowdoin Buoy, (C) CARIACO, (D) MVCO, (E) Palmer, (F) Plumes and Blooms. Pigment loadings mirror the order and color of the cluster results for each observatory. Pigments are colored by major taxonomic group: cyanobacteria (light blue), haptophytes (dark blue), diatoms (brown), dinoflagellates (red), green algae (green), cryptophytes (purple), crysophytes (gold). Suggested taxonomic affiliation of pigments that are either positively and negatively correlated with Modes 1-4 are indicated. The mode number and percent variance explained by that mode are listed above each plot. Numbers above each pigment represent the correlation coefficient of that pigment with the given mode multiplied by 100.

Table S1 uploaded separately.

Table S1. Summary of cruise names, dates, HPLC processing facility, and pigment/environmental data source for all cruises included in this analysis. Data were

processed at Horn Point Laboratory (HPL), NASA Goddard Space Flight Center (NASA GSFC), Laboratoire Oceanographique de Villefranche (LOV), the Australian Commonwealth Scientific and Industrial Research Organisation (CSIRO), the Alfred Wegner Institute (AWI), and the DiTullio lab at the College of Charleston. Detailed data citations given, indicated by superscripts in the table.

Source	Fuco	Perid	19but	19hex	Allo	MVchlb	Zea
All	0.29	0.03	0.04	0.12	0.01	0.04	0.04
HPL	0.26	0.04	**0.10***	0.06	0.02	0.05	0.07
GSFC	0.24	0.02	0.02	0.08	0.01	0.05	0.03
LOV	0.10	0.03	**0.01**	**0.05**	0.01	0.04	0.03
AWI	0.24	0.02	0.03	0.10	0.01	0.04	**0.08***
CSIRO	**0.06**	**0.01**	0.02	0.08	0.01	**0.03**	0.04
DiTullio	**0.54***	**0.06**	0.03	**0.24***	0.02	0.05	0.03

Table S2. Mean value of biomarker pigments in the global dataset for all six source labs. HPL = Horn Point Labs, GSFC = NASA Goddard Space Flight Center, LOV = Laboratoire d'Océanographie de Villefranche-sur-Mer, AWI = Alfred Wegner Institute, CSIRO = Commonwealth Scientific and Industrial Research Organisation, DiTullio = DiTullio lab (College of Charleston). Bold values indicate the highest (red) and lowest (blue) values for each parameter. If the highest or lowest value is the same for a given pigment, the value is not indicated in bold or color. A star indicates that the value was significantly different from the mean values of that pigment for all other labs (2-way ANOVA, $p<0.001$).

Supporting Information for How can phytoplankton pigments be best used to characterize surface ocean phytoplankton groups for ocean color remote sensing algorithms?

Authors: Sasha J. Kramer and David A. Siegel

Supplementary Table 1. Summary of cruise names, dates, HPLC processing facility, and pigment/environmental data source for all cruises included in this analysis. Data were processed at Horn Point Laboratory (HPL), NASA Goddard Space Flight Center (NASA GSFC), Laboratoire Oceanographique de Villefranche (LOV), the Australian Commonwealth Scientific and Industrial Research Organisation (CSIRO), the Alfred Wegner Institute (AWI), and the DiTullio lab at the College of Charleston. Detailed data citations given below, indicated by superscripts in the table. All data are available at https://doi.pangaea.de/10.1594/PANGAEA.905883.

Dataset	Cruise(s)	# data pts.	Dates	Processed @	Region	Pigment data source	Environmental data source
AMMA[1]		9	2006	HPL	Equatorial Atlantic Ocean	SeaBASS	NOAA NODC
AMT[2]	19	91	2009	HPL	Atlantic Ocean	SeaBASS	BODC
POLARSTERN	XXIII1[3], XXIV1[4], XXIV4[5], XXV1[6], XXVI4[7], XXVIII3[8]	441	2005-2012	AWI	Global	SeaBASS/PANGAEA	PANGAEA
DiTullio	CoFeMUG[9], TRACERS[10], CORSACS[11]	861	2005-2006, 2011	DiTullio	Southern Ocean	BCO-DMO	BCO-DMO/PANGAEA/pers. comm.

BEAGLE[12]	Legs 1, 2, 4, 5, 6	183	2003	CMAR	Global, 30°S	CSIRO	US Hydro/JAMEST
BIOSOPE[13]		73	2004	LOV	Equatorial Pacific Ocean	pers. comm	LOV
CLIVAR	A16N[14], I06S[14], I08S[14], I09N[14], P16S[14], P16N[14], P18[14], S04[15]	430	2007-2016	HPL/GSFC	Global	SeaBASS	US Hydro
CliVEC[16]	4	76	2011	GSFC	Northeast U.S. shelf	SeaBASS	NOAA NESFC
CSIRO[17]	BROKEWEST, SAZSENSE, SS012004, SS032006, SS072004, SS092004, SS092010	205	2003-2010	CMAR	Coastal Oceania, Southern Ocean	AEsOP	Australian Antarctic Data Centre/Australian Marine National Facility
Lohrenz	Cape Hatteras[18], GOMECC[19], MagMix1[20], MagMix2[21]	186	2008-2010	HPL	Gulf of Mexico	SeaBASS	pers. comm./Joung & Schiller (2016)
ICESCAPE[22]	1,2	188	2010-2011	GSFC	Arctic Ocean	SeaBASS	pers. comm.

LineP[23]		13	2009	HPL	Northeast Pacific Ocean	SeaBASS	Line P Program
Malina[24]		171	2009	LOV	Arctic Ocean	LOV	pers. comm.
NAAMES[25]	1,2,3,4	205	2015-2018	GSFC	Northwest Atlantic Ocean	SeaBASS	NOAA PMEL
NABE 08[26]		44	2008	HPL	North Atlantic Ocean	SeaBASS	BCO-DMO
MSM and SONNE	M91[27], MSM183[28], MSM91[29], SO202[30], SO218[31]	366	2002-2012	AWI	Global	SeaBASS/PANGAEA / pers. comm.	PANGAEA
Peru[32]		307	2000	DiTullio	Peru Upwelling Zone	MAREDAT	pers. comm.
POMME[33]	1,2,3	34	2001	LOV	Northeast Atlantic Ocean	MAREDAT	pers. comm.
RemSens POC[34]	AE1319, NH1418	81	2013, 2014	GSFC	Atlantic and Pacific Oceans	SeaBASS	SAMOS

SABOR[35]		44	2014	GSFC	Northwest Atlantic Ocean	SeaBASS	pers. comm.
SBI[36]	1,2	59	2004	HPL	Arctic Ocean	SeaBASS	UCAR/NCAR, Arctic Data Center
SOCCOM[37]	P15S, A12, SR1B, NBP1701, P17E, P182017	32	2014-2017	GSFC	Southern Ocean	SeaBASS	US Hydro
TAO[38]/EqBox06[39]		139	2006, 2012	HPL, GSFC	Equatorial Pacific Ocean	SeaBASS	pers. comm.
Tara	Tara Oceans 2009-2013[40], Tara Polar[41], Tara Mediterranean[42]	242	2009-2016	LOV	Global	SeaBASS	PANGAEA/pers. comm.
Palmer LTER[43]	January cruises	155	2013-2016	Rutgers	West Antarctic Peninsula	Environmental Data Initiative	N/A
Martha's Vineyard Coastal Observatory (MVCO)[44]	Monthly cruises	278	2004-2014	HPL, GSFC	Martha's Vineyard, MA, USA	SeaBASS	N/A
Bowdoin Buoy[45]	Monthly cruises, April-October	161	2008-2016	GSFC	Harpswell Sound, ME, USA	SeaBASS	N/A

Plumes and Blooms[46]	Monthly cruises	711	2005-2017	HPL, GSFC	Santa Barbara Channel, CA, USA	SeaBASS	N/A
BOUSSOLE[47]	Monthly cruises	221	2001-2006	LOV	Mediterranean Sea	SeaBASS	N/A
CARIACO[48]	Monthly cruises	81	2006-2014	HPL, GSFC	Cariaco Basin	SeaBASS	N/A

Dataset Citations

1. AMMA
   - Nelson, Norman (2006). African Monsoon Multidisciplinary Analyses Program (AMMA). SeaWiFS Bio-optical Archive and Storage System (SeaBASS), NASA. Accessed: 24 August 2017. http://dx.doi.org/10.5067/SeaBASS/AMMA/DATA001
   - R. D. Castle; Wanninkhof, R.; J. Shannahoff (2011). Partial pressure (or fugacity) of carbon dioxide, salinity and other variables collected from Surface underway observations using Barometric pressure sensor, Carbon dioxide (CO2) gas analyzer and other instruments from NOAA Ship RONALD H. BROWN in the Caribbean Sea, Coastal Waters of Florida and others from 2006-02-16 to 2006-12-02 (NODC Accession 0081021). Version 3.3. National Oceanographic Data Center, NOAA. Dataset. Accessed 09 October 2017.

2. AMT
   - Balch, William and Dall'Olmo, Giorgio (2009). Atlantic Meridional Transect (AMT) Cruise 19. SeaWiFS Bio-optical Archive and Storage System (SeaBASS), NASA. Accessed: 24 August 2017. http://dx.doi.org/110.5067/SeaBASS/AMT/DATA001
   - Biological and Hydrographic station data collected during the Atlantic Meridional Transect (AMT) programme (1995-present), RRS James Cook cruise JC039 (AMT19). Published by the British Oceanographic Data Centre, National Environment Research Council, United Kingdom. https://www.bodc.ac.uk/data/documents/series/1760659

3. POLARSTERN XXIII/1

- Bracher, Astrid; Taylor, Marc H; Taylor, Bettina B; Dinter, Tilman; Röttgers, Rüdiger; Steinmetz, Francois (2015): Phytoplankton pigment concentrations during POLARSTERN cruise ANT-XXIII/1. PANGAEA, https://doi.org/10.1594/PANGAEA.871713, In supplement to: Bracher, A et al. (2015): Using empirical orthogonal functions derived from remote-sensing reflectance for the prediction of phytoplankton pigment concentrations. *Ocean Science*, 11(1), 139-158, https://doi.org/10.5194/os-11-139-2015.
- Budéus, Gereon (2007): Physical oceanography measured on water bottle samples during POLARSTERN cruise ANT-XXIII/1. Alfred Wegener Institute, Helmholtz Center for Polar and Marine Research, Bremerhaven, PANGAEA, https://doi.org/10.1594/PANGAEA.633678.

4. POLARSTERN XXIV/1
   - Bracher, Astrid (2015): Phytoplankton pigment concentrations during POLARSTERN cruise ANT-XXIV/1. Alfred Wegener Institute, Helmholtz Center for Polar and Marine Research, Bremerhaven, PANGAEA, https://doi.org/10.1594/PANGAEA.848583, In supplement to: Bracher, Astrid; Taylor, Marc H; Taylor, Bettina B; Dinter, Tilman; Röttgers, Rüdiger; Steinmetz, Francois (2015): Using empirical orthogonal functions derived from remote-sensing reflectance for the prediction of phytoplankton pigment concentrations. *Ocean Science*, 11(1), 139-158, https://doi.org/10.5194/os-11-139-2015.
   - Schnack-Schiel, Sigrid B; Rohardt, Gerd (2009): Continuous thermosalinograph oceanography along POLARSTERN cruise track ANT-XXIV/1. Alfred Wegener Institute, Helmholtz Center for Polar and Marine Research, Bremerhaven, PANGAEA, https://doi.org/10.1594/PANGAEA.727463.

5. POLARSTERN XXIV/4
   - Bracher, Astrid (2015): Phytoplankton pigment concentrations during POLARSTERN cruise ANT-XXIV/4. Alfred Wegener Institute, Helmholtz Center for Polar and Marine Research, Bremerhaven, PANGAEA, https://doi.org/10.1594/PANGAEA.848584, In supplement to: Bracher, Astrid; Taylor, Marc H; Taylor, Bettina B; Dinter, Tilman; Röttgers, Rüdiger; Steinmetz, Francois (2015): Using empirical orthogonal functions derived from remote-sensing reflectance for the prediction of phytoplankton pigment concentrations. *Ocean Science*, 11(1), 139-158, https://doi.org/10.5194/os-11-139-2015.
   - Schmitt, Bettina; Rohardt, Gerd (2010): Physical oceanography during POLARSTERN cruise ANT-XXIV/4. Alfred Wegener Institute, Helmholtz Center for Polar and Marine Research, Bremerhaven, PANGAEA, https://doi.org/10.1594/PANGAEA.742855.

6. POLARSTERN XXV/1

- Taylor, Bettina B; Torrecilla, Elena; Bernhardt, Anja; Taylor, Marc H; Peeken, Ilka; Röttgers, Rüdiger; Piera, Jaume; Bracher, Astrid (2011): Pigments of phytoplankton during POLARSTERN cruise ANT-XXV/1. PANGAEA, https://doi.org/10.1594/PANGAEA.819070, In supplement to: Taylor, BB et al. (2011): Bio-optical provinces in the eastern Atlantic Ocean and their biogeographical relevance. *Biogeosciences*, 8(12), 3609-3629, https://doi.org/10.5194/bg-8-3609-2011
- Kattner, Gerhard; Rohardt, Gerd (2010): Physical oceanography during POLARSTERN cruise ANT-XXV/1. Alfred Wegener Institute, Helmholtz Center for Polar and Marine Research, Bremerhaven, PANGAEA, https://doi.org/10.1594/PANGAEA.744706.

7. POLARSTERN XXVI/4
   - Bracher, Astrid (2015): Phytoplankton pigment concentrations during POLARSTERN cruise ANT-XXVI/4. Alfred Wegener Institute, Helmholtz Center for Polar and Marine Research, Bremerhaven, PANGAEA, https://doi.org/10.1594/PANGAEA.848585, In supplement to: Bracher, Astrid; Taylor, Marc H; Taylor, Bettina B; Dinter, Tilman; Röttgers, Rüdiger; Steinmetz, Francois (2015): Using empirical orthogonal functions derived from remote-sensing reflectance for the prediction of phytoplankton pigment concentrations. *Ocean Science*, 11(1), 139-158, https://doi.org/10.5194/os-11-139-2015
   - Rohardt, Gerd; Bracher, Astrid (2011): Physical oceanography during POLARSTERN cruise ANT-XXVI/4. Alfred Wegener Institute, Helmholtz Center for Polar and Marine Research, Bremerhaven, PANGAEA, https://doi.org/10.1594/PANGAEA.758127

8. POLARSTERN XXVIII/3
   - Bracher, Astrid (2014): Phytoplankton pigment concentrations during POLARSTERN cruise ANT-XXVIII/3. Alfred Wegener Institute, Helmholtz Center for Polar and Marine Research, Bremerhaven, PANGAEA, https://doi.org/10.1594/PANGAEA.848588, In supplement to: Soppa, Mariana A; Hirata, Takafumi; Silva, Brenner; Dinter, Tilman; Peeken, Ilka; Wiegmann, Sonja; Bracher, Astrid (2014): Global retrieval of diatom abundance based on phytoplankton pigments and satellite data. *Remote Sensing*, 6(10), 10089-10106, https://doi.org/10.3390/rs61010089
   - Strass, Volker H; Leach, Harry; Prandke, Hartmut; Donnelly, Matthew; Bracher, Astrid; Wolf-Gladrow, Dieter A (2016): Physical oceanography during POLARSTERN cruise ANT-XXVIII/3. PANGAEA, https://doi.org/10.1594/PANGAEA.840334, In supplement to: Strass, VH et al. (2016): The physical environmental conditions for biogeochemical differences along the Antarctic Circumpolar Current in the Atlantic Sector during late austral summer 2012. *Deep Sea Research Part II: Topical Studies in Oceanography*, 20 pp, https://doi.org/10.1016/j.dsr2.2016.05.018

9. CoFeMUG
   - DiTullio, Giacomo (2011). HPLC analyses of algal pigment concentrations from the CoFeMUG cruise (KN192-05) in the South Atlantic subtropical gyre during 2007. Biological and Chemical Oceanography Data Management Office (BCO-DMO). Dataset version 2011-05-18. https://www.bco-dmo.org/dataset/3480/data. Accessed 17 September 2017.
   - Environmental data from Mak Saito, personal communication

10. TRACERS
   - DiTullio, Giacomo (2015) Sample HPLC pigments from RVIB Nathaniel B. Palmer NBP1302 cruise in the Ross Sea during 2013 (TRACERS project). Biological and Chemical Oceanography Data Management Office (BCO-DMO). Dataset version 2015-05-18. https://www.bco-dmo.org/dataset/558908/data. Accessed 17 September 2017.
   - Hansell, D., (2014). Underway Hydrographic, Weather and Ship-state Data (JGOFS) from Nathaniel B. Palmer expedition NBP1302 (2013). Integrated Earth Data Applications (IEDA). doi: http://dx.doi.org/10.1594/IEDA/320062

11. CORSACS
   - DiTullio, Giacomo (2010). Underway data including temperature, salinity, fluorometry, pigments from RVIB Nathaniel B. Palmer NBP0601 cruise in the Ross Sea Southern Ocean (CORSACS project). Biological and Chemical Oceanography Data Management Office (BCO-DMO). Dataset version 2010-09-09. https://www.bco-dmo.org/dataset/3366/data. Accessed 17 September 2017.
   - DiTullio, Giacomo (2010). Algal pigment concentrations as measured by HPLC from RVIB Nathaniel B. Palmer cruises in the Ross Sea Southern Ocean from 2005 to 2006 (CORSACS project). Biological and Chemical Oceanography Data Management Office (BCO-DMO). Dataset version 2010-09-08. https://www.bco-dmo.org/dataset/3360/data. Accessed 17 September 2017.
   - Long, Matthew C; Dunbar, Robert B; Tortell, Philippe Daniel; Smith, Walker O Jr; Mucciarone, David A; DiTullio, Giacomo R (2011): (Supplement table S1) Physical oceanography of summer transect stations sampled during Nathaniel B. Palmer cruise NBP06-01, Ross Sea. PANGAEA, https://doi.org/10.1594/PANGAEA.818032, In supplement to: Long, MC et al. (2011): Vertical structure, seasonal drawdown, and net community production in the Ross Sea, Antarctica. *Journal of Geophysical Research*, 116(C10), C10029, https://doi.org/10.1029/2009JC005954.
   - Long, Matthew C; Dunbar, Robert B; Tortell, Philippe Daniel; Smith, Walker O Jr; Mucciarone, David A; DiTullio, Giacomo R (2011): (Supplement table S1) Physical oceanography of spring transect stations sampled during Nathaniel B. Palmer cruise NBP06-08, Ross Sea. PANGAEA, https://doi.org/10.1594/PANGAEA.818031, In supplement to: Long, MC et al. (2011): Vertical structure, seasonal drawdown, and net community production in the Ross Sea, Antarctica. *Journal of Geophysical Research*, 116(C10), C10029, https://doi.org/10.1029/2009JC005954.

12. BEAGLE
- Australian-waters Earth Observation Phytoplankton-type products (AEsOP) project. Acessed: 14 September 2017. http://aesop.csiro.au
- Physical and chemical data were obtained from the online CLIVAR database (https://cchdo.ucsd.edu/). We thank the NSF/NOAA-funded U.S. CLIVAR Repeat Hydrography Program (funding provided by the NOAA Climate Program Office and the NOAA Atlantic Oceanographic and Meteorological Laboratory).
- Japan Agency for Marine-Earth Science and Technology (http://www.jamstec.go.jp/iorgc/ocorp/data/beagle2003/index.html).

13. BIOSOPE
- Ras, Joséphine and Claustre, Hervé (2004). BIogeochemistry and Optics South Pacific Experiment (BIOSOPE). Accessed 24 August 2017. http://www.obs-vlfr.fr/proof/vt/op/ec/biosope/bio_obj.htm
- Loic Petit de la Villeon (2004). BIogeochmistry and Optics South Pacific Experiment (BIOSOPE). Accessed 08 October 2017. http://www.obs-vlfr.fr/proof/vt/op/ec/biosope/bio_obj.htm

14. CLIVAR A16N, I06S, I08S, I09N, P16S, P16N, P18
- Nelson, Norman and Siegel, David (2003-2016). Climate Variability and Predictability (CLIVAR). SeaWiFS Bio-optical Archive and Storage System (SeaBASS), NASA. Accessed: 24 August 2017 and 12 December 2018. http://dx.doi.org/10.5067/SeaBASS/CLIVAR/DATA001
- Physical and chemical data were obtained from the online CLIVAR database (https://cchdo.ucsd.edu/). We thank the NSF/NOAA-funded U.S. CLIVAR Repeat Hydrography Program (funding provided by the NOAA Climate Program Office and the NOAA Atlantic Oceanographic and Meteorological Laboratory).

15. CLIVAR S04
- McClain, Charles and Neeley, Aimee (2013). Climate Variability and Predictability (CLIVAR). SeaWiFS Bio-optical Archive and Storage System (SeaBASS), NASA. Accessed: 24 August 2017. http://dx.doi.org/10.5067/SeaBASS/CLIVAR/DATA001
- Physical and chemical data were obtained from the online CLIVAR database (https://cchdo.ucsd.edu/). We thank the NSF/NOAA-funded U.S. CLIVAR Repeat Hydrography Program (funding provided by the NOAA Climate Program Office and the NOAA Atlantic Oceanographic and Meteorological Laboratory).

16. CLIVEC

- Mannino, Anthony (2011). The Impacts of Climate Variability on Primary Productivity and Carbon Distributions in the Middle Atlantic Bight and Gulf of Maine (CliVEC). SeaWiFS Bio-optical Archive and Storage System (SeaBASS), NASA. Accessed: 24 August 2017. http://dx.doi.org/10.5067/SeaBASS/CLIVEC/DATA001
- NOAA NEFSC (2011). NOAA Northeast Fisheries Science Center's DEL1004 ECOMON/NASA Ground Truth Measurements Survey. SeaWiFS Bio-optical Archive and Storage System (SeaBASS), NASA. Accessed: 20 October 2017. http://dx.doi.org/10.5067/SeaBASS/NOAA_NEFSC/DEL1004_CV4

17. CSIRO AEsOP
- Australian-waters Earth Observation Phytoplankton-type products (AEsOP) project. Acessed: 14 September 2017. http://aesop.csiro.au
- Rosenberg, M., Gorton, R. (2006, updated 2016) BROKE West Survey, Marine Science Cruise AU0603 - Oceanographic Field Measurements and Analysis Australian Antarctic Data Centre - CAASM Metadata (https://data.aad.gov.au/metadata/records/BROKE-West_CTD_au0603).
- Howard, W., Griffiths (Retired), B. (2006, updated 2016) Physical and biogeochemical dynamics of the subantarctic zone Australian Antarctic Data Centre - CAASM Metadata (https://data.aad.gov.au/metadata/records/ASAC_2720).
- Wiley, P. (2006, updated 2014) Aurora Australis Voyage 3 2005-2006 (BROKE-West) Underway Data Australian Antarctic Data Centre - CAASM Metadata (https://data.aad.gov.au/metadata/records/200506030).

18. GOMEX
- Lohrenz, Steven (2010). Measurements made near Cape Hatteras. SeaWiFS Bio-optical Archive and Storage System (SeaBASS), NASA. Accessed: 24 August 2017. http://dx.doi.org/10.5067/SeaBASS/GOMECC/DATA001
- Environmental data from Steve Lohrenz, personal communication

19. Cape Hatteras
- Lohrenz, Steven and Mannino, Anthony (2012). The Gulf of Mexico and East Coast Carbon Cruise (GOMECC). SeaWiFS Bio-optical Archive and Storage System (SeaBASS), NASA. Accessed: 24 August 2017. http://dx.doi.org/10.5067/SeaBASS/CAPEHATTERAS2010/DATA001
- Environmental data from Steve Lohrenz, personal communication

20. MagMix 1
- Lohrenz, Steven (2008). MagMix1. SeaWiFS Bio-optical Archive and Storage System (SeaBASS), NASA. Accessed: 24 August 2017. http://dx.doi.org/10.5067/SeaBASS/MAGMIX/DATA001
- Environmental data from Steve Lohrenz, personal communication

21. MagMix 2
- Lohrenz, Steven (2009). MagMix2. SeaWiFS Bio-optical Archive and Storage System (SeaBASS), NASA. Accessed: 12 December 2018. http://dx.doi.org/10.5067/SeaBASS/MAGMIX/DATA001
- Joung, D., & Shiller, A. M. (2016). Temporal and spatial variations of dissolved and colloidal trace elements in Louisiana Shelf waters. *Marine Chemistry*, 181, 25-43.

22. ICESCAPE
- Laney, Sam (2010-2011). Impacts of Climate on the Eco-Systems and Chemistry of the Arctic Pacific Environment (ICESCAPE). SeaWiFS Bio-optical Archive and Storage System (SeaBASS), NASA. Accessed: 24 August 2017. http://dx.doi.org/10.5067/SeaBASS/ICESCAPE/DATA001
- Environmental data from Gert van Dijken, personal communication

23. Line P
- Westberry, Toby (2009). Measurements taken along a transect of the Gulf of Alaska and Northeastern Pacific Ocean. SeaWiFS Bio-optical Archive and Storage System (SeaBASS), NASA. Accessed: 24 August 2017. http://dx.doi.org/10.5067/SeaBASS/LINE_P/DATA001
- Line P Program, Cruise 2009-10 (https://waterproperties.ca/linep/2009-10/index.php#Data).

24. Malina
- Ras, Joséphine and Claustre, Hervé (2009). Malina Project. Accessed 16 October 2017. http://www.obs-vlfr.fr/proof/php/malina/x_datalist_1.php?xxop=malina&xxcamp=malina
- Gratton, Yves, Prieur, Louis, and Tremblay, Bruno (2009). Malina Project. Accessed 16 October 2017. http://www.obs-vlfr.fr/proof/php/malina/x_datalist_1.php?xxop=malina&xxcamp=malina
- CTD environmental data from Tim Papakyriakou and Colline Gombault, personal communication
- Underway environmental data from Amundsen Science Data Collection. TSG data collected by the CCGS Amundsen in the Canadian Arctic. 2009. ArcticNet Inc., Québec, Canada. Raw data. Limited distribution. Accessed from PDC@arcticnet.ulaval.ca, on November 21, 2017.

25. NAAMES 1-4
- Behrenfeld, Michael and Graff, Jason (2015-2017). North Atlantic Aerosols and Marine Ecosystems Study (NAAMES). SeaWiFS Bio-optical Archive and Storage System (SeaBASS), NASA. Accessed: 24 August 2017 and 12 December 2018. http://dx.doi.org/10.5067/SeaBASS/NAAMES/DATA001

- Nelson, Norm and Siegel, David (2015-2017), North Atlantic Aerosols and Marine Ecosystems Study (NAAMES). SeaWiFS Bio-optical Archive and Storage System (SeaBASS), NASA. Accessed: 24 August 2017 and 12 December 2018. http://dx.doi.org/10.5067/SeaBASS/NAAMES/DATA001
- NAAMES 4 HPLC: Jason Graff, personal communication; Norm Nelson, personal communication
- NAAMES 1. PMEL Atmospheric Chemistry Data Server. https://saga.pmel.noaa.gov/data/PrePlot.php?cruise=NAAMES1. Accessed October 16, 2017.
- NAAMES 2. PMEL Atmospheric Chemistry Data Server. https://saga.pmel.noaa.gov/data/PrePlot.php?cruise=NAAMES2. Accessed October 16, 2017.
- NAAMES 3. PMEL Atmospheric Chemistry Data Server. https://saga.pmel.noaa.gov/data/PrePlot.php?cruise=NAAMES3. Accessed December 12, 2018.
- NAAMES 4. PMEL Atmospheric Chemistry Data Server. https://saga.pmel.noaa.gov/data/PrePlot.php?cruise=NAAMES4. Accessed December 12, 2018.

26. NABE08
    - Perry, Mary Jane and Westberry, Toby (2008). Measurements made in the North Atlantic Bight. SeaWiFS Bio-optical Archive and Storage System (SeaBASS), NASA. Accessed: 24 August 2017. http://dx.doi.org/10.5067/SeaBASS/NAB08/DATA001
    - Perry, Mary Jane (2011) Niskin bottle hydrography from the CTD rosette from cruises KN193-03, B4-2008, B9-2008, and B10-2008 from the subpolar North Atlantic and Iceland Basin in 2008 (NAB 2008 project). Biological and Chemical Oceanography Data Management Office (BCO-DMO). Dataset version 2011-05-10. http://lod.bco-dmo.org/id/dataset/3393. Accessed October 16, 2017.

27. METEOR M91
    - Hepach, Helmke; Quack, Birgit; Tegtmeier, Susann; Engel, Anja; Bracher, Astrid; Fuhlbrügge, Steffen; Galgani, Luisa; Atlas, Elliot L; Lampel, Johannes; Frieß, Udo; Krüger, Kirstin (2016): Pigment measured on water bottle samples during METEOR cruise M91. PANGAEA, https://doi.org/10.1594/PANGAEA.864786, In supplement to: Hepach, H et al. (2016): Biogenic halocarbons from the Peruvian upwelling region as tropospheric halogen source. *Atmospheric Chemistry and Physics*, 16(18), 12219-12237, https://doi.org/10.5194/acp-16-12219-2016.
    - Krahmann, Gerd; Bange, Hermann W (2016): Physical oceanography during METEOR cruise M91. Research Center for Marine Geosciences at Christian Albrechts University, Kiel, PANGAEA, https://doi.pangaea.de/10.1594/PANGAEA.858090

28. MSM18/3

- Bracher, Astrid (2015): Phytoplankton pigment concentrations during Maria S. Merian cruise MSM18/3. Alfred Wegener Institute, Helmholtz Center for Polar and Marine Research, Bremerhaven, PANGAEA, https://doi.org/10.1594/PANGAEA.848586, In supplement to: Bracher, Astrid; Taylor, Marc H; Taylor, Bettina B; Dinter, Tilman; Röttgers, Rüdiger; Steinmetz, Francois (2015): Using empirical orthogonal functions derived from remote-sensing reflectance for the prediction of phytoplankton pigment concentrations. *Ocean Science*, 11(1), 139-158, https://doi.org/10.5194/os-11-139-2015
- Krahmann, Gerd; Fischer, Tim (2012): Physical oceanography during Maria S. Merian cruise MSM18/3. IFM-GEOMAR Leibniz-Institute of Marine Sciences, Kiel University, PANGAEA, https://doi.org/10.1594/PANGAEA.783445, In supplement to: Brandt, Peter; Bange, Hermann W; Banyte, Donata; Dengler, Marcus; Didwischus, Sven-Helge; Fischer, Tim; Greatbatch, Richard J; Hahn, Johannes; Kanzow, Torsten; Karstensen, Johannes; Körtzinger, Arne; Krahmann, Gerd; Schmidtko, Sunke; Stramma, Lothar; Tanhua, Toste; Visbeck, Martin (2015): On the role of circulation and mixing in the ventilation of oxygen minimum zones with a focus on the eastern tropical North Atlantic. *Biogeosciences*, 12(2), 489-512, https://doi.org/10.5194/bg-12-489-2015

29. MSM9/1
- Bracher, Astrid; Taylor, Bettina B (2017): Phytoplankton pigment concentrations measured by HPLC during Maria S. Merian cruise MSM9/1. Alfred Wegener Institute, Helmholtz Center for Polar and Marine Research, Bremerhaven, PANGAEA, https://doi.org/10.1594/PANGAEA.873070
- Mertens, Christian; Rhein, Monika; Walter, Maren; Böning, Claus W; Behrens, Erik; Kieke, Dagmar; Steinfeldt, Reiner; Stöber, Uwe (2014): Physical oceanography during Maria S. Merian cruise MSM09/1. PANGAEA, https://doi.org/10.1594/PANGAEA.871382, In supplement to: Mertens, C et al. (2014): Circulation and transports in the Newfoundland Basin, western subpolar North Atlantic. *Journal of Geophysical Research-Oceans*, 119(11), 7772-7793, https://doi.org/10.1002/2014JC010019.

30. SONNE SO2/02
- Taylor, Bettina B; Bracher, Astrid (2017): Pigment concentrations measured in surface water during SONNE cruise SO202/2 (TRANSBROM). PANGAEA, https://doi.org/10.1594/PANGAEA.880235, In supplement to: Zindler, Cathleen; Bracher, Astrid; Marandino, Christa A; Taylor, B; Torrecilla, Elena; Kock, Annette; Bange, Hermann W (2013): Sulphur compounds, methane, and phytoplankton: interactions along a north-south transit in the western Pacific Ocean. *Biogeosciences*, 10(5), 3297-3311, https://doi.org/10.5194/bg-10-3297-2013
- Zindler, Cathleen; Bange, Hermann W; Marandino, Christa A (2013): Underway measurements of DMS, DMSP and DMSO during SONNE cruise 202/2 (TRANSBROM). PANGAEA, https://doi.org/10.1594/PANGAEA.805613, In supplement to: Zindler, Cathleen; Bracher, Astrid; Marandino, Christa A; Taylor, B; Torrecilla, Elena; Kock, Annette;

Bange, Hermann W (2013): Sulphur compounds, methane, and phytoplankton: interactions along a north-south transit in the western Pacific Ocean. *Biogeosciences*, 10(5), 3297-3311, https://doi.org/10.5194/bg-10-3297-2013.

31. SONNE SO2/18
   - Bracher, Astrid (2014): Phytoplankton pigments measured on water bottle samples during SONNE cruise SO218. Alfred Wegener Institute, Helmholtz Center for Polar and Marine Research, Bremerhaven, PANGAEA, https://doi.org/10.1594/PANGAEA.848589, In supplement to: Soppa, Mariana A; Hirata, Takafumi; Silva, Brenner; Dinter, Tilman; Peeken, Ilka; Wiegmann, Sonja; Bracher, Astrid (2014): Global retrieval of diatom abundance based on phytoplankton pigments and satellite data. *Remote Sensing*, 6(10), 10089-10106, https://doi.org/10.3390/rs61010089.
   - Krahmann, Gerd; Quack, Birgit (2015): Physical oceanography during SONNE cruise SO218. PANGAEA, https://doi.org/10.1594/PANGAEA.841411

32. Peru
   - Buitenhuis, E. T., Vogt, M., Moriarty, R., Bednarsek, N., Doney, S. C., Leblanc, K., C. Le Quéré et al. (2013). MAREDAT: towards a world atlas of MARine Ecosystem DATa. Earth System Science Data, 5(2), 227.
   - Environmental data from Ken Bruland, personal communication

33. POMME
   - Prieur, Louis, Ras, Joséphine, and Claustre, Hervé (2001). Programme Océan Multidisciplinaire Méso Echelle (POMME) in Buitenhuis, E. T., Vogt, M., Moriarty, R., Bednarsek, N., Doney, S. C., Leblanc, K., C. Le Quéré et al. (2013). MAREDAT: towards a world atlas of MARine Ecosystem DATa. Earth System Science Data, 5(2), 227.
   - Environmental data from Louis Prieur, personal communication

34. RemSens POC
   - Cetinić, Ivona (2013). RemSens POC. SeaWiFS Bio-optical Archive and Storage System (SeaBASS), NASA. Accessed: 10 December 2018. http://dx.doi.org/10.5067/SeaBASS/REMSENSPOC/DATA001
   - Cetinić, Ivona (2014). RemSens POC. SeaWiFS Bio-optical Archive and Storage System (SeaBASS), NASA. Accessed: 10 December 2018. http://dx.doi.org/10.5067/SeaBASS/REMSENSPOC/DATA001
   - Smith, S. R., J. J. Rolph, K. Briggs, M. A. Bourassa, 2013: Quality Controlled Shipboard Automated Meteorological and Oceanographic System (SAMOS) Data. Center for Ocean-Atmospheric Prediction Studies, The Florida State University, Tallahassee, FL, USA. http://samos.coaps.fsu.edu. Accessed 12 December 2018.

- Smith, S. R., J. J. Rolph, K. Briggs, M. A. Bourassa, 2014: Quality Controlled Shipboard Automated Meteorological and Oceanographic System (SAMOS) Data. Center for Ocean-Atmospheric Prediction Studies, The Florida State University, Tallahassee, FL, USA. http://samos.coaps.fsu.edu. Accessed 12 December 2018.

35. SABOR
    - Behrenfeld, Michael and Twardowski, Michael (2014). Ship-Aircraft Bio-Optical Research (SABOR). SeaWiFS Bio-optical Archive and Storage System (SeaBASS), NASA. Accessed: 24 August 2017. http://dx.doi.org/10.5067/SeaBASS/SABOR/DATA001
    - Environmental data from Ivona Cetinić, personal communication

36. SBI
    - Hill, Victoria (2004). Western Arctic Shelf-Basin Interactions (SBI) experiment. SeaWiFS Bio-optical Archive and Storage System (SeaBASS), NASA. Accessed: 24 August 2017 and 12 December 2018. http://dx.doi.org/10.5067/SeaBASS/SBI/DATA001
    - Bates, N., Benner, R., Best, M., Hansell, D., Hill, V., Kirchman, D., Roberts, S., Service Group, Scripps Institution of Oceanography, University of California - San Diego. 2012. SBI Cruise HLY0402 merged bottle dataset. Version 1.0. UCAR/NCAR - Earth Observing Laboratory. https://doi.org/10.5065/D6DF6PB5. Accessed 17 Oct 2017.
    - Nick R. Bates, Ronald Benner, Margaret H. P. Best, Dennis Hansell, Victoria J. Hill, et al. SBI Cruise HLY0403 merged bottle dataset. Arctic Data Center. http://dx.doi.org/10.5065/D6SF2T8T. Accessed 11 Dec 2018.

37. SOCCOM: P15S, A12, SR1B, NBP1701, P17E, P18/2017
    - Boss, Emmanuel and Talley, Lynne (2015-2017). Southern Ocean Carbon and Climate Observations and Modeling project (SOCCOM). SeaWiFS Bio-optical Archive and Storage System (SeaBASS), NASA. Accessed: 24 August 2017. http://dx.doi.org/10.5067/SeaBASS/SOCCOM/DATA001
    - Physical and chemical data were obtained from the online CLIVAR database (https://cchdo.ucsd.edu/). We thank the NSF/NOAA-funded U.S. CLIVAR Repeat Hydrography Program (funding provided by the NOAA Climate Program Office and the NOAA Atlantic Oceanographic and Meteorological Laboratory).

38. TAO
    - Behrenfeld, Michael (2012). Measurements from the ships visiting the TAO (Tropical Atmosphere Ocean). SeaWiFS Bio-optical Archive and Storage System (SeaBASS), NASA. Accessed: 24 August 2017. http://dx.doi.org/10.5067/SeaBASS/TAO/DATA001
    - Environmental data from Wayne Slade, personal communication

39. EqBox

- Behrenfeld, Michael, Boss, Emmanuel, and Nelson, Norman (2006). Measurements from the ships visiting the TAO (Tropical Atmosphere Ocean). SeaWiFS Bio-optical Archive and Storage System (SeaBASS), NASA. Accessed: 24 August 2017. http://dx.doi.org/10.5067/SeaBASS/TAO/DATA001
- Environmental data from Wayne Slade, personal communication

40. Tara Oceans

- Boss, Emmanuel and Claustre, Hervé (2009-2012). Tara Oceans Expedition. SeaWiFS Bio-optical Archive and Storage System (SeaBASS), NASA. Accessed: 24 August 2017. http://dx.doi.org/10.5067/SeaBASS/TARA_OCEANS_EXPEDITION/DATA001
- Picheral, Marc; Searson, Sarah; Taillandier, Vincent; Bricaud, Annick; Boss, Emmanuel; Ras, Josephine; Claustre, Hervé; Ouhssain, Mustapha; Morin, Pascal; Tremblay, Jean-Éric; Coppola, Laurent; Gattuso, Jean-Pierre; Metzl, Nicolas; Thuillier, Doris; Gorsky, Gabriel; Tara Oceans Consortium, Coordinators; Tara Oceans Expedition, Participants (2014): Vertical profiles of environmental parameters measured on discrete water samples collected with Niskin bottles during the Tara Oceans expedition 2009-2013. PANGAEA, https://doi.org/10.1594/PANGAEA.836319.
- Boss, Emmanuel; Picheral, Marc; Searson, Sarah; Le Goff, Hervé; Reverdin, Gilles; Leeuw, Thomas; Chase, Alison; Bricaud, Annick; Kolber, Zbigniew S; Taillandier, Vincent; Pesant, Stephane; Tara Oceans Consortium, Coordinators; Tara Oceans Expedition, Participants (2017): Underway surface water data during the Tara Oceans expedition in 2009-2012. PANGAEA, https://doi.org/10.1594/PANGAEA.873566, In: Boss, Emmanuel; Picheral, Marc; Searson, Sarah; Marec, Claudie; Le Goff, Hervé; Reverdin, Gilles; Leeuw, Thomas; Chase, Alison; Anderson, Leif G; Gattuso, Jean-Pierre; Pino, Diana Ruiz; Padín, Xose Antonio; Grondin, Pierre-Luc; Matuoka, Atsushi; Babin, Marcel; Bricaud, Annick; Kolber, Zbigniew S; Taillandier, Vincent; Hafez, Mark; Chekalyuk, Alexander; Pesant, Stephane; Météo France; Tara Oceans Consortium, Coordinators (2017): Harmonised data from underway navigation, meteorology and surface water measurements during the Tara Oceans expedition in 2009-2013. PANGAEA, https://doi.org/10.1594/PANGAEA.873592.

41. Tara Polar

- Babin, Marcel and Boss, Emmanuel (2013). Tara Oceans Polar Circle. SeaWiFS Bio-optical Archive and Storage System (SeaBASS), NASA. Accessed: 24 August 2017. http://dx.doi.org/10.5067/SeaBASS/TARA_OCEANS_POLAR_CIRCLE/DATA001
- Boss, Emmanuel; Picheral, Marc; Searson, Sarah; Marec, Claudie; Le Goff, Hervé; Reverdin, Gilles; Leeuw, Thomas; Chase, Alison; Anderson, Leif G; Gattuso, Jean-Pierre; Pino, Diana Ruiz; Gyllencreutz, Richard; Grondin, Pierre-Luc; Matuoka, Atsushi; Babin, Marcel; Bricaud, Annick; Taillandier, Vincent; Hafez, Mark; Chekalyuk, Alexander; Pesant,

Stephane (2017): Underway surface water data during the Tara Oceans expedition in 2013. PANGAEA, https://doi.org/10.1594/PANGAEA.873567, In: Boss, Emmanuel; Picheral, Marc; Searson, Sarah; Marec, Claudie; Le Goff, Hervé; Reverdin, Gilles; Leeuw, Thomas; Chase, Alison; Anderson, Leif G; Gattuso, Jean-Pierre; Pino, Diana Ruiz; Padín, Xose Antonio; Grondin, Pierre-Luc; Matuoka, Atsushi; Babin, Marcel; Bricaud, Annick; Kolber, Zbigniew S; Taillandier, Vincent; Hafez, Mark; Chekalyuk, Alexander; Pesant, Stephane; Météo France; Tara Oceans Consortium, Coordinators (2017): Harmonised data from underway navigation, meteorology and surface water measurements during the Tara Oceans expedition in 2009-2013. PANGAEA, https://doi.org/10.1594/PANGAEA.873592.

42. Tara Mediterranean
    - Boss, Emmanuel and Claustre, Hervé (2014). Tara Mediterranean. SeaWiFS Bio-optical Archive and Storage System (SeaBASS), NASA. Accessed: 12 December 2018. http://dx.doi.org/10.5067/SeaBASS/Tara_Med/DATA001
    - Environmental data from Emmanuel Boss and Alison Chase, personal communication

43. Palmer LTER
    - Schofield O.. 2018. Photosynthetic pigments of water column samples and analyzed with High Performance Liquid Chromatography (HPLC), collected aboard Palmer LTER annual cruises off the coast of the Western Antarctica Peninsula, 2009-2016. Environmental Data Initiative. https://doi.org/10.6073/pasta/4d583713667a0f52b9d2937a26d0d82e. Dataset accessed 5/05/2019.

44. Martha’s Vineyard Coastal Observatory
    - Sosik, Heidi (2004-2014). Martha's Vineyard Coastal Observatory (MVCO). SeaWiFS Bio-optical Archive and Storage System (SeaBASS), NASA. Accessed: 18 June 2015. http://dx.doi.org/10.5067/SeaBASS/MVCO/DATA001

45. Bowdoin Buoy
    - Roesler, Collin (2008-2016). Bowdoin Buoy. SeaWiFS Bio-optical Archive and Storage System (SeaBASS), NASA. Accessed: 20 June 2017. http://dx.doi.org/10.5067/SeaBASS/BOWDOINBUOY/DATA001

46. Plumes and Blooms
    - Siegel, David (2005-2017). Plumes and Blooms. SeaWiFS Bio-optical Archive and Storage System (SeaBASS), NASA. Accessed: 24 August 2017. http://dx.doi.org/10.5067/SeaBASS/PLUMES_AND_BLOOMS/DATA001

47. BOUSSOLE

- Antoine, David (2001-2006). BOUée pour l'acquiSition d'une Séries Optique à Long termE (BOUSSOLE). SeaWiFS Bio-optical Archive and Storage System (SeaBASS), NASA. Accessed: 24 August 2017. http://dx.doi.org/10.5067/SeaBASS/BOUSSOLE/DATA001

48. CARIACO
- Muller-Karger, Frank (2006-2014). CArbon Retention In A Colored Ocean (CARIACO). SeaWiFS Bio-optical Archive and Storage System (SeaBASS), NASA. Accessed: 17 September 2017. http://dx.doi.org/10.5067/SeaBASS/CARIACO/DATA001

Supplementary Table 1 Acknowledgements

We wish to thank all researchers and crew members involved with data collection, preparation, analysis, and submission for collecting this data and making it available for use. When requested, we have included specific information about funding or limits to data use here. Thank you to Stefanie Schumacher and Amelie Driemel at PANGAEA for support with data table uploads and formatting.

- All funding for NSF/NOAA U.S. CLIVAR Repeat Hydrography Program provided by the NOAA Climate Program Office and the NOAA Atlantic Oceanographic and Meteorological Laboratory.
- All datasets from CSIRO were collected on voyages on the RV Southern Surveyor granted by the Marine National Facility. They are made available under a Creative Commons Attribution 4.0 International License.
- The acquisition of BIOSOPE data was funded through Centre National de Recherche Scientifique (CNRS) & Institut National des Sciences de l’Univers (INSU) grants.
- Some of the data presented herein were collected by the Canadian research icebreaker CCGS Amundsen and made available by the Amundsen Science program, which is supported by the Canada Foundation for Innovation Major Science Initiatives Fund. The views expressed in this publication do not necessarily represent the views of Amundsen Science or that of its partners.
